# Bacteria-powered LA@CaDGP biomotor: a multi-modal weapon integrating calcium overload, chemotherapy, and starvation for breast cancer therapy

**DOI:** 10.1186/s12951-025-03968-w

**Published:** 2026-01-06

**Authors:** Jingrong Huang, Yongcheng Tang, Kewei Xiang, Biqiong Wang, Jia Wang, Yun Lu, Yue Li, Hongjun Deng, Tao Li, Kang Xiong, Qinglian Wen, Shaozhi Fu

**Affiliations:** 1 Department of Oncology, The Affiliated Hospital, Southwest Medical University, Luzhou, 646000 Sichuan PR China; 2 Department of General Surgery (Hepatopancreatobiliary Surgery), The Affiliated Hospital, Southwest Medical University, Luzhou, 646000 Sichuan PR China; 3 Medicine and health Klinikum rechts der Isar, Technical University of Munich, Ismaningerstr. 22, 81675 München, Germany; 4 Department of Radiation Oncology, Cancer Center, West China Hospital, Sichuan University, Chengdu, 610041 Sichuan PR China; 5 Nuclear Medicine and Molecular Imaging Key Laboratory of Sichuan Province, Luzhou, 646000 Sichuan PR China

**Keywords:** Breast cancer, Calcium carbonate nanoparticles, Lactobacillus acidophilus, Doxorubicin, Glucose oxidase, Starvation therapy

## Abstract

Chemotherapy remains a cornerstone in breast cancer treatment, but poor drug targeting compromises its efficacy and exacerbates side effects. To optimize drug delivery, we developed a novel bacteria-propelled biomotor system, designated as LA@CaDGP, to enhance the tumor-specific drug delivery. The biomotor was engineered to load doxorubicin (DOX) and glucose oxidase (GOD) within mesoporous calcium carbonate nanoparticles (CaCO_3_ NPs), which are conjugated to Lactobacillus acidophilus (L. *acidophilus*, LA) via a polydopamine (PDA) coating. Following tumor accumulation facilitated by bacterial tropism, the CaCO_3_ component undergoes dissolution, releasing calcium ions that induce mitochondrial dysfunction and thereby potentiate the chemotherapeutic efficacy of DOX. Concurrently, the GOD-mediated glucose depletion effect synergistically enhances antitumor activity through metabolic intervention. In a mouse orthotopic breast cancer model, the LA@CaDGP group showed a tenfold higher DOX concentration in tumor tissues compared to conventional free DOX administration, while the DOX concentration in heart tissues was 24 times lower. Mice in the LA@CaDGP group achieved a median survival time of 50 days. Collectively, these findings collectively demonstrate that the LA@CaDGP biomotor constitutes a promising therapeutic platform for breast cancer, integrating multiple synergistic mechanisms: calcium overload-mediated cytotoxicity, conventional chemotherapy, and metabolic starvation therapy.

**Graphical Abstract:**

**Figure:**
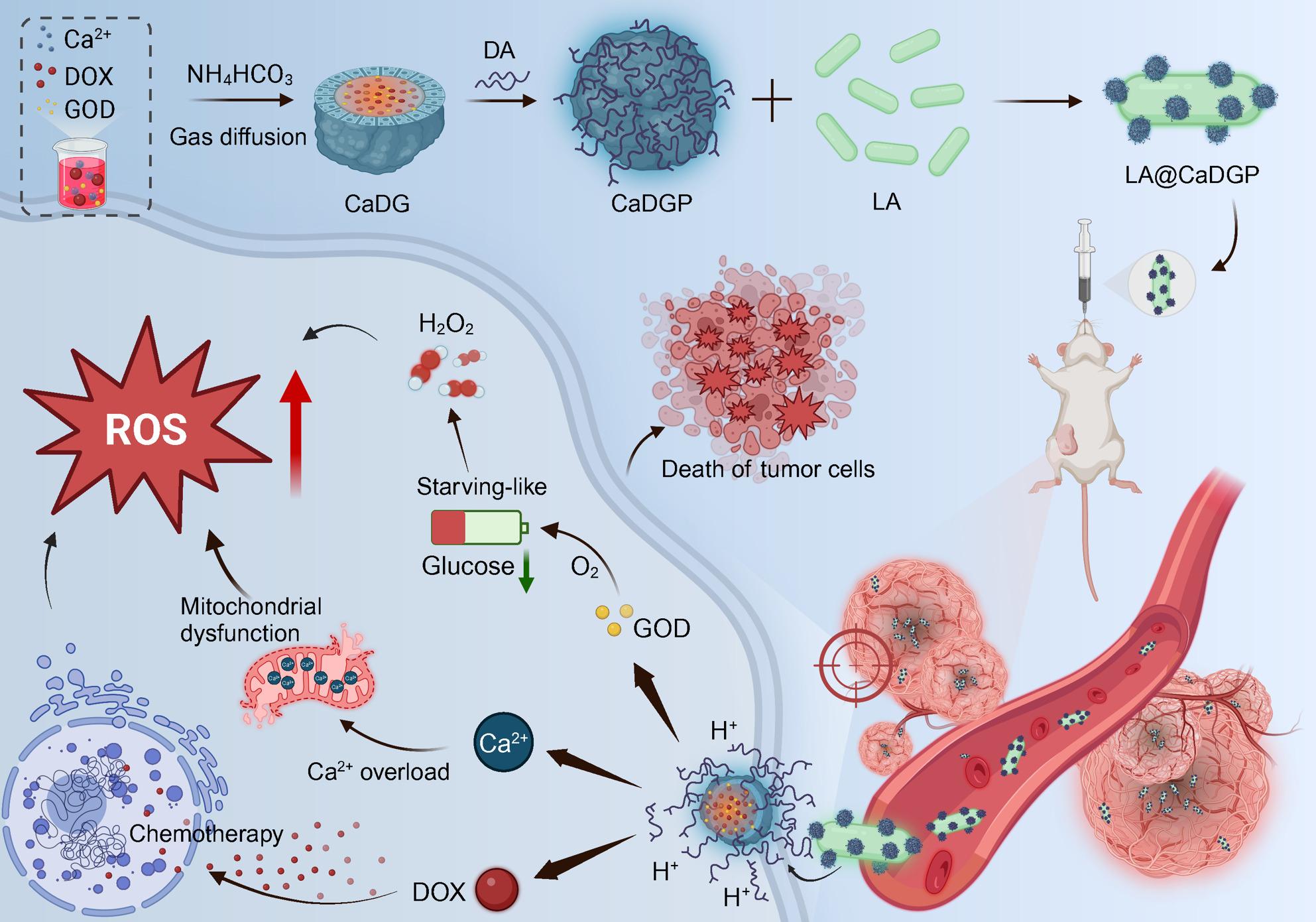
In this study, an oral self-propelled biomotor called LA@CaDGP was designed to simultaneously deliver the chemotherapeutic drug doxorubicin and glucose oxidase for a combination of chemotherapy and starvation therapy to fight breast cancer. These biomotors actively gathered in the tumor and achieved improved anti-tumor effects by directly killing tumor cells, depleting cellular nutrients, and boosting calcium overload.

**Supplementary Information:**

The online version contains supplementary material available at 10.1186/s12951-025-03968-w.

## Introduction

Breast cancer is the most prevalent type of cancer among females in China [1]. Its clinical management requires a comprehensive approach, including surgical resection, chemotherapy, radiotherapy, endocrine therapy, and targeted drug therapy to improve patient survival rates [2]. Chemotherapy, as a primary treatment modality, demonstrates significant efficacy but is often accompanied by substantial toxic effects due to its limited ability to specifically target tumors [3]. Extensive research has shown that the tumor microenvironment (TME) plays a fundamental role in both the initiation and progression of cancer, while also significantly hindering the uptake of chemotherapeutic drugs [4, 5]. Due to distinct vascular barriers and a hypoxic TME, traditional chemotherapy formulations face significant challenges in efficiently reaching tumor tissues through the bloodstream [6], resulting in severe adverse effects, such as cardiotoxicity, hepatotoxicity, and nephrotoxicity [7, 8]. Therefore, the development of novel formulations with enhanced targeting capabilities is essential to overcome the limitations of conventional drug delivery in terms of efficiency and selectivity [9].

Doxorubicin, a commonly used chemotherapy drug, primarily exerts its effects by intercalating into the DNA base pairs of cancer cells, thereby blocking DNA replication and RNA transcription [10]. However, the effectiveness of single-agent chemotherapy is often limited [11] and can lead to drug resistance and systemic toxicity [12]. Recent research indicates that the combining chemotherapy with starvation therapy notably enhances therapeutic outcomes for breast cancer [13]. Glucose oxidase (GOD), a key component of starvation therapy [14], catalyzes the conversion of glucose into hydrogen peroxide (H_2_O_2_) and glucuronic acid, thereby depleting the tumor’s energy supply and inducing localized oxidative stress [15]. However, GOD is susceptible to inactivation in the bloodstream due to advanced catalytic reactions and proteolysis [16, 17], highlighting the importance of loading GOD into a suitable carrier to prevent loss of activity [18]. Nanocarriers are effective in binding, absorbing, and transporting small-molecule drugs [19, 20, 21]. They accumulate at tumor sites via the bloodstream and release the drug molecules for therapeutic effect [22]. Calcium carbonate (CaCO_3_) nanoparticles, known for their biocompatibility and ease of preparation, can respond intelligently to the TME [23], releasing Ca^2+^ ions that damage tumor cell mitochondria [24]. The disruption of the mitochondrial membrane leads to the release of Ca^2+^ and apoptosis factors into the cytoplasm, promoting the formation of reactive oxygen species (ROS) [25]. Furthermore, mitochondrial damage leads to decreased ATP levels and reduced HIF-1α expression, thereby enhancing the susceptibility of tumor cells to chemotherapy [26]. The potential of anaerobic probiotics to target solid tumors has been widely recognized in recent years [27, 28]. Lactobacillus acidophilus (L. acidophilus, LA), a naturally occurring anaerobic probiotic in the human body, exhibits excellent biocompatibility and actively colonizes hypoxic tumor cores. It can be employed as a living micromotor [29]. Utilizing LA as a carrier enables precise drug delivery and localized release, increasing drug concentration at tumor-site while reducing systemic toxicity [30]. Therefore, drug delivery systems based on CaCO_3_ nanospheres not only enhance drug stability in the bloodstream, minimize toxic effects on normal tissue cells, improve drug penetration into tumor tissue, and extend retention time [31], but also synergistically enhance the efficacy of chemotherapy drugs [32].

**Fig. 1:**
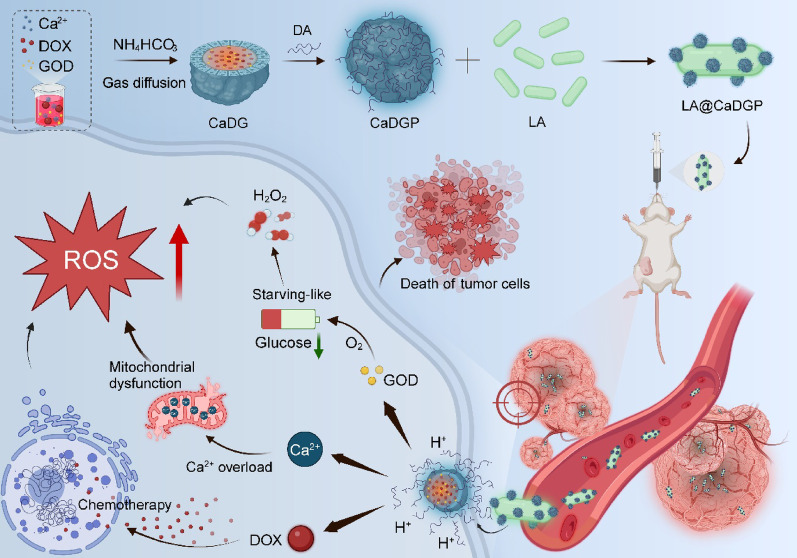
Schematic illustration showing the fabrication process and antitumor mechanism of the biohybrid LA@CaDGP

In our research, we designed a bioactive nanodrug delivery platform (LA@CaDGP) to target TME. As illustrated in Fig. 1, DOX and GOD were first encapsulated in mesoporous CaCO_3_ nanospheres, which were subsequently coated with polydopamine (PDA) and attached to the surface of Lactobacillus acidophilus (L. acidophilus, LA). Once these biomotors are absorbed into the bloodstream, they actively target and colonize hypoxic tumor tissues. The CaCO_3_ nanoparticles bound to the bacterial surface are then released into the TME in response to the antioxidant glutathione (GSH) [33]. In the acidic environment, they react with H^+^ ions to generate Ca^2+^ ions and CO_2_, thereby accelerating drug release to facilitate lysosomal escape and enhancing DOX-induced tumor cell death via calcium overload [34, 35]. Furthermore, due to the decreased expression of catalase, H_2_O_2_ accumulates in cancer cells, resulting in elevated oxidative stress in TME [36]. Consequently, we established an in situ mouse model of breast cancer to evaluate the anti-tumor efficacy of the developed biomotors, LA@CaDGP.

## Results

### Preparation and characterization of CaDGP NPs

**Fig. 2:**
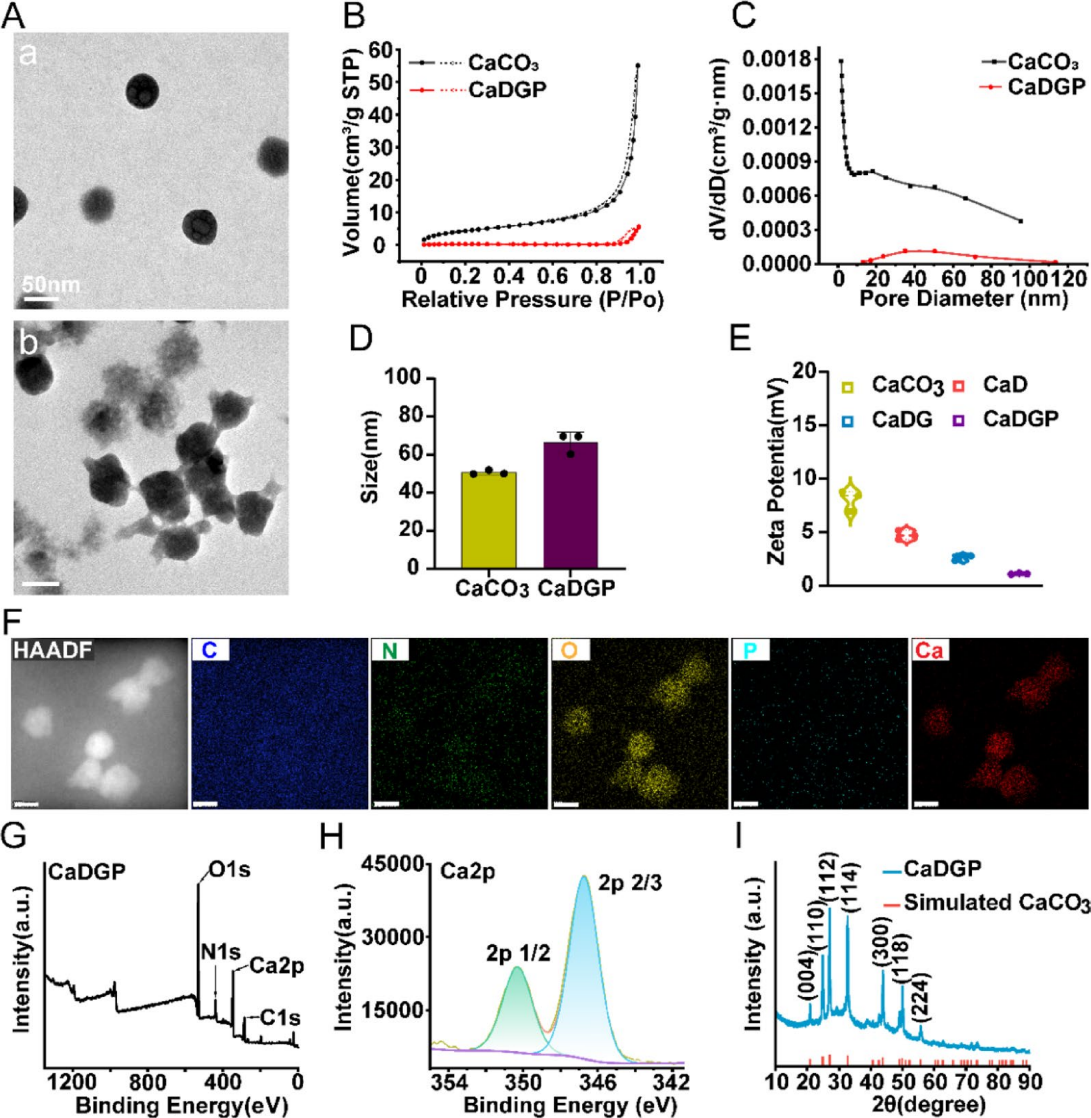
Preparation and characterization of various drug formulations. (**A**) TEM images of (a) CaCO_3_ NPs and (b) CaDGP NPs, scale bar: 50 nm. (**B**) Nitrogen adsorption/desorption isotherms of CaCO_3_ NPs and CaDGP NPs. (**C**) The corresponding pore-size distribution curve of CaCO_3_ NPs and CaDGP NPs. (**D**) Average diameters of CaCO_3_ NPs and CaDGP NPs. (**E**) Zeta potentials of CaCO_3_ NPs, CaD NPs, CaDG NPs, and CaDGP NPs. (**F**) HAADF-STEM image and elemental mapping of CaDGP NPs, scale bar: 50 nm. (**G**) XPS spectrum of CaDGP NPs. (**H**) XPS spectrum of Ca 2p in the CaDGP NPs. (**I**) XRD patterns of CaDGP NPs. Abbreviations indicated as follows: CaD (CaCO_3_ + DOX); CaDG (CaCO_3_ + DOX + GOD); CaDGP (CaCO_3_ + DOX + GOD + PDA)

CaCO_3_ nanoparticles were synthesized by a one-pot method and then used as carriers to load DOX and GOD. TEM images (Fig. 2Aa-b) show that the prepared CaCO_3_ NPs were spherical, whereas the CaDGP NPs appeared as viscous, round-like particles. The nitrogen adsorption-desorption isotherms (Fig. 2B) demonstrate that the CaCO_3_ NPs exhibited a mesoporous structure. After loading with DOX and GOD, notable reductions in specific surface area (from 16.2870 to 0.8001m^2^/g) and pore volume (from 0.083448 to 0.008561cm^3^/g) were observed. (Fig. 2C), indicating the excellent absorption capacity of the CaCO_3_ NPs. DLS results (Fig. 2D) show that the average diameter of the CaDGP NPs was approximately 66.44 ± 3.11 nm, which was slightly larger than that of the CaCO_3_ NPs (50.63 ± 1.18 nm), consistent with the TEM results. As shown in Fig. 2E, the average zeta potential of the CaCO_3_ NPs decreased from 8.05 ± 0.97 mV to 1.12 ± 0.05 mV after loading DOX and GOD to produce CaDGP NPs. The prepared nanoparticles with different drug loadings exhibited various colors (Fig. S1).

To further verify the successful preparation of CaDGP NPs, elemental mapping was first conducted. As shown in Fig. 2F and Fig. S2, the elements Ca, O, N, and P were uniformly distributed within the CaDGP nanoparticles. Here, N is derived from doxorubicin and polydopamine, while P is a characteristic element of GOD. XPS analysis confirmed that CaDGP NPs contained four main elements: Ca, O, N, and C (Fig. 2G). The high-resolution XPS spectrum (Fig. 2H) shows binding energy peaks for Ca 2p₃/₂ and Ca 2p₁/₂ at 346.4 eV and 350.1 eV, respectively, indicating the presence of Ca in the form of Ca^2+^ ions. XRD analysis (Fig. 2I) shows that the diffraction peaks of CaDGP NPs matched the standard peaks of CaCO_3_, suggesting that the structure of CaCO_3_ NPs was not altered during the synthesis process of CaDGP NPs. In Fig. S3, the UV-Vis spectra of CaDGP NPs show characteristic absorption peaks for DOX (480 nm) and GOD (275 nm), indicating the successful encapsulation of both components within the CaCO_3_ NPs. FT-IR spectroscopy further confirms the presence of GOD-specific bands in CaDGP NPs (Fig. S4): Amide I at 1637 cm⁻¹ (C = O stretch) and Amide II at 1542 cm⁻¹ (N − H bend/C − N stretch coupling). These spectral signatures provide strong evidence for the incorporation of GOD into the CaDGP NPs [37]. According to the standard curve shown in Fig. S5, the GOD loading content was 6.53 ± 0.74%. The encapsulation efficiencies (EE) and drug loading (DL) of DOX in the CaDGP NPs were 77.36 ± 0.19% and 14.41 ± 0.14%, respectively.

**Fig. 3:**
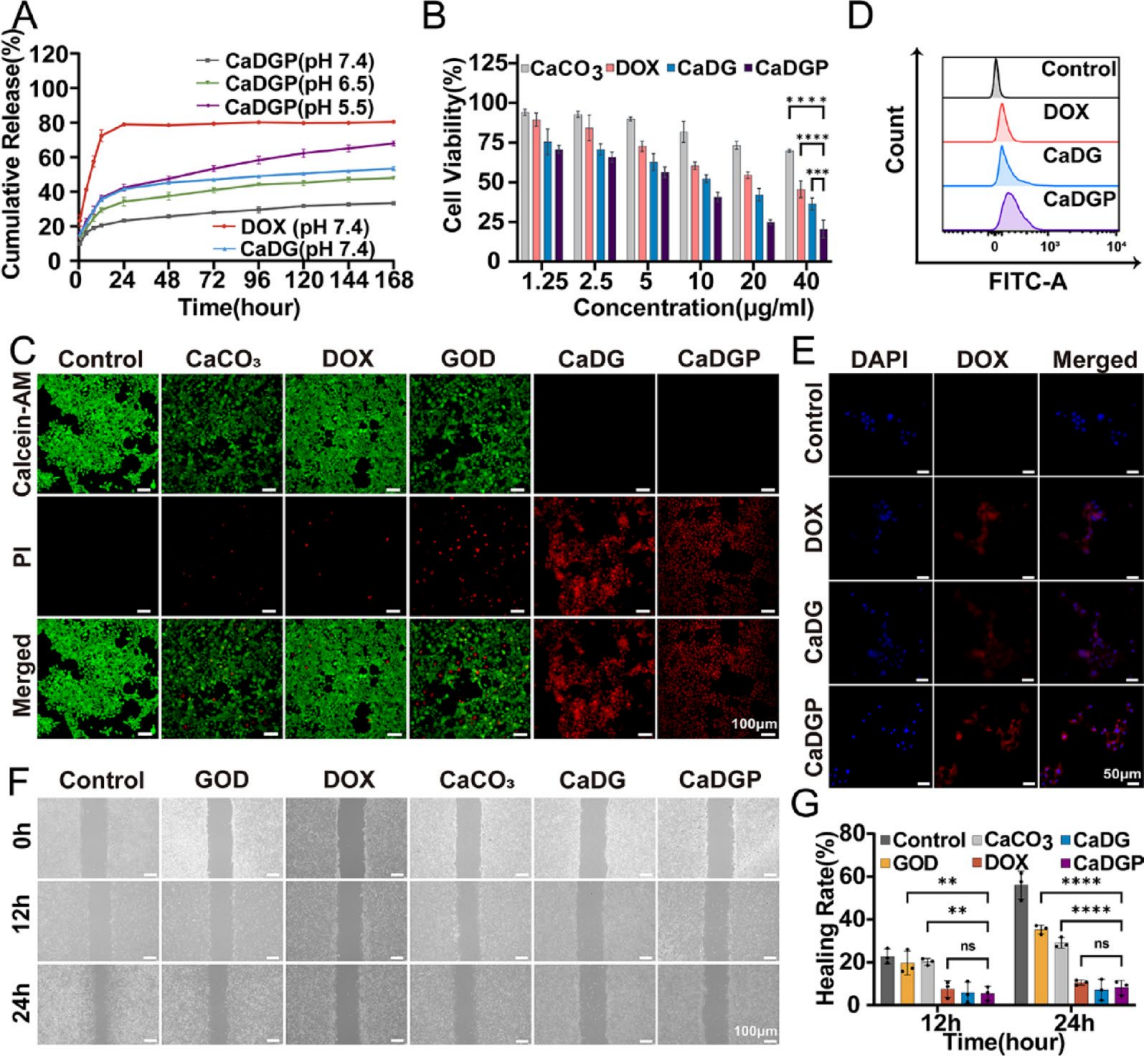
DOX release and cellular toxicity, uptake, and inhibition of cell migration of various drugs. (**A**) In vitro release profiles of free DOX, CaDG, and CaDGP in PBS at pH 5.5, 6.5, and 7.4 (*n* = 3). (**B**) Cell viability of the CaCO_3_, DOX, CaDG, and CaDGP nanoparticles on 4T1 cells at pH 6.5 (*n* = 3). (**C**) Live/dead staining of 4T1 cells with different treatments for 24 h: Calcein-AM (green, live cells), PI (red, dead cells), scale bar: 100 μm. (**D**) Quantitative analysis of DOX fluorescence intensity within A549 cells. (**E**) The fluorescence images of various drugs taken up by A549 cells, scale bar: 50 μm. (**F**) Photographs of wound healing at 0, 12, and 24 h. (**G**) The healing rate of scratch test (*n* = 3), scale bar:100 μm. Results are presented as means ± SD. ns: no statistical significance, **P < 0.01, ***P < 0.001, ****P < 0.0001

To verify the responsiveness of CaDGP NPs to the acidic TME, the release behavior of DOX was investigated in PBS adjusted to neutral and acidic conditions (Fig.  3A). At pH 7.4, the release of DOX from CaDG NPs was less than 55%, which was lower than that from free DOX (79.03%), reflecting the encapsulation effect of calcium carbonate. The release of DOX from CaDGP NPs was even lower (33.35%) due to the presence of the PDA coating. In contrast, at pH 5.5, the cumulative release percentage of DOX from CaDGP NPs reached approximately 45.25% within 48 h and gradually increased to 68.01% by the seventh day, indicating that DOX release from CaDGP NPs was enhanced under the acidic conditions characteristic of the tumor microenvironment. 

GOD has been employed to enhance multimodal cancer therapy through its ability to catalyze the conversion of glucose to gluconic acid, which results in a significant pH reduction [38]. To verify the catalytic functionality of GOD encapsulated in CaCO_3_ NPs, we quantitatively monitored pH changes in glucose solutions containing various NPs. As shown in Fig. S6, free GOD rapidly acidified the glucose solution (pH 7.4 to 3.89), while immobilized GOD in CaDG NPs showed slower kinetics (pH 7.4 to 5.56). This demonstrates that CaCO_3_ dissolved due to the acid produced during GOD-catalyzed glucose decomposition. Notably, the pH of the CaCO_3_ group (~ 7.55) and the CaDGP group (~ 7.38) remained near neutral, confirming that the PDA coating delayed the release of GOD.

### In vitro cellular toxicity, uptake, and inhibition of cell migration

The results of the MTT assay are shown in Fig. S7-S8 and Fig. 3B. At pH 7.4, the CaCO_3_ NPs exhibited minimal cytotoxicity, with cell viability remaining above 80% even at a concentration of 40 µg/mL (Fig. S7). The cell viability in the CaDGP group was 5% higher than that in the DOX group, which may be attributed to the slow release caused by the PDA coating. At pH 6.5 (Fig. 3B), cell viability in the CaDGP group decreased to approximately 20%, indicating a significantly enhanced in vitro anti-tumor effect of CaDGP NPs in an acidic environment. As shown in Fig. S8, the DOX formulations exhibited similar cytotoxic effects on both A549 and CT26 cells. Further confirmation of the cytotoxic effects of the various drugs was obtained through Calcein-AM/PI staining of the three tumor cell types (Fig. 3 C and Fig. S9-S10). The results showed a higher number of PI-positive cells in the CaDGP group compared to the free DOX group.

The uptake of DOX by tumor cells (4T1, A549, and CT26) was quantitatively analyzed using flow cytometry. As shown in Fig. 3D and Fig. S11, cellular internalization of DOX was higher in the CaDGP NPs than in the free DOX. Moreover, these findings were consistent with the fluorescence images shown in Fig. 3E and Fig. S12. Significant red fluorescence of DOX was observed in the DOX, CaDG, and CaDGP groups, indicating that CaDGP NPs were effectively taken up by tumor cells. The photos of the scratch test are presented in Fig.  3F and G. After 24 h of incubation, the wound healing rate for 4T1 cells in the Control group (56.02%) was markedly higher than those in the GOD (35.33%), CaCO_3_ (29.11%), and DOX groups (10.61%). Notably, the CaDG and CaDGP groups showed even lower healing rates, at 7.16% and 8.28%, respectively, demonstrating a strong inhibitory effect on cell migration. The same trend was observed in A549 cells (Fig. S13).

### Calcium overload induced cell destruction

**Fig. 4:**
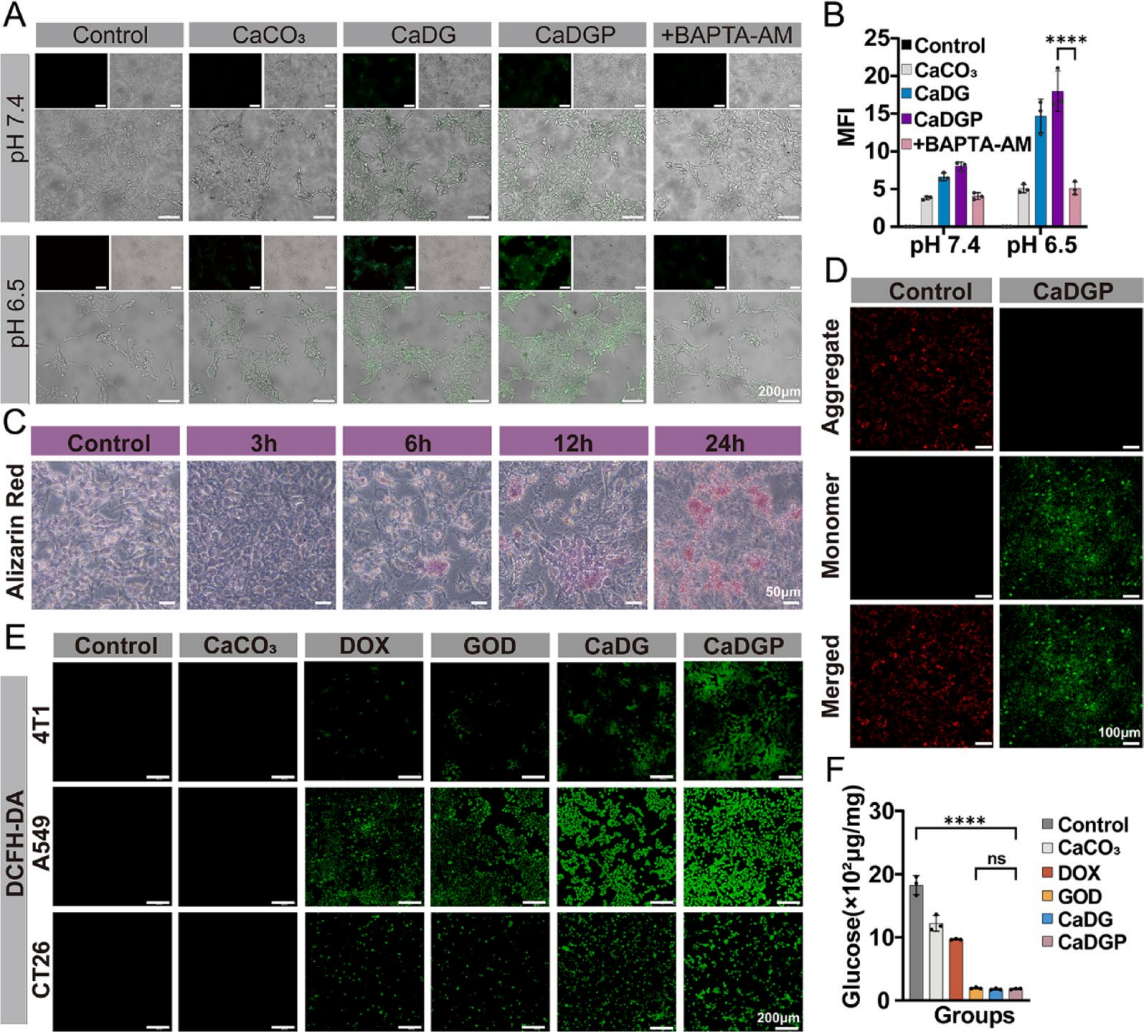
Calcium overload and oxidative stress in vitro. (**A**) The fluorescence images of intracellular uptake of Ca^2+^ in 4T1 cells after different treatments at pH 7.4 and 6.5 (scale bar: 200 μm), and the quantitative fluorescence intensity of each group (**B**) (*n* = 3). (**C**) Identification of exocytosis products of 4T1 cells via Alizarin Red staining (scale bar: 50 μm). (**D**) Fluorescence images of JC-1-stained 4T1 cells (scale bar: 100 μm). (**E**) Fluorescence images of DCFH-DA assays of 4T1cells, A549 cells and CT26 cells under different treatments (scale bar: 200 μm). (**F**) Glucose content in 4T1 cells with indicated treatments (*n* = 3). Results are presented as means ± SD. ns: no statistical significance, ****P < 0.0001

The Ca²⁺-sensitive dye Fluo-4 AM was used as a probe to observe the uptake behavior of the Ca-containing drugs. Once the probe enters tumor cells, it is metabolized to Fluo-4, which can bind to Ca²⁺ and emit green fluorescence. As demonstrated in Fig. 4A and B and Fig. S14-S15, due to the homeostatic mechanisms maintained by tumor cells, undecorated CaCO_3_ NPs were only weakly absorbed, resulting in minimal fluorescence observed within the cells. In contrast, CaDGP NPs exhibited significant cellular internalization, producing strong green fluorescence. The stronger green fluorescence was observed in CaDGP NPs at pH 6.5, indicating excellent responsiveness to the TME. However, when the calcium chelator BAPTA-AM was added, fluorescence in the CaDGP group decreased significantly, suggesting that treatment with CaDGP NPs led to a substantial increase in intracellular Ca²⁺ levels. This elevation of intracellular calcium is crucial for disrupting the structural functions of tumor cells. Alizarin Red staining was also employed to examine Ca²⁺ accumulation within tumor cells (Fig. 4C and Fig. S16). The increasing red calcification area over time indicates that the mitochondrial damage may be associated with Ca²⁺ metabolic dysfunction. When nanoparticles are internalized into tumor cells via endocytosis, they are typically trapped in endosomes and eventually delivered to acidic lysosomes, which can limit their therapeutic effectiveness against tumors. Therefore, facilitating the escape of the cargo from lysosomal vesicles is important to enhance efficacy [39]. As shown in Fig. S17, CaDGP treatment significantly reduced the red fluorescence intensity in cells, indicating altered lysosomal membrane permeability. This suggests that CaDGP NPs facilitated intracellular escape by disrupting lysosomal function, thereby preventing premature drugs degradation [40].

JC-1 staining was performed to evaluate the mitochondrial function of tumor cells. As shown in Fig. 4D and Fig. S18, the fluorescence colors reflected changes in mitochondrial membrane potential and revealed disruption of physiological functions. After the mitochondrial membrane is damaged, substances diffuse into the cytoplasm, leading to increased ROS generation, which ultimately results in the death of tumor cells. The DCFH-DA probe was used to assess ROS generation, as intracellular ROS reacts with the probe to emit green fluorescence. From the fluorescence images (Fig. 4E) and the quantitative results (Fig. S19), it is evident that 4T1 cells in the CaDGP group exhibited the highest green fluorescence intensity compared to the free DOX and GOD groups, indicating significant ROS production due to the combination of multiple therapies. Furthermore, as shown in Fig. 4F and Fig. S20, quantitative analysis of glucose content within tumor cells demonstrated that the CaDGP group successfully induced a starvation effect compared to the free GOD group.

### In vitro bioactivity of LA@CaDGP

**Fig. 5:**
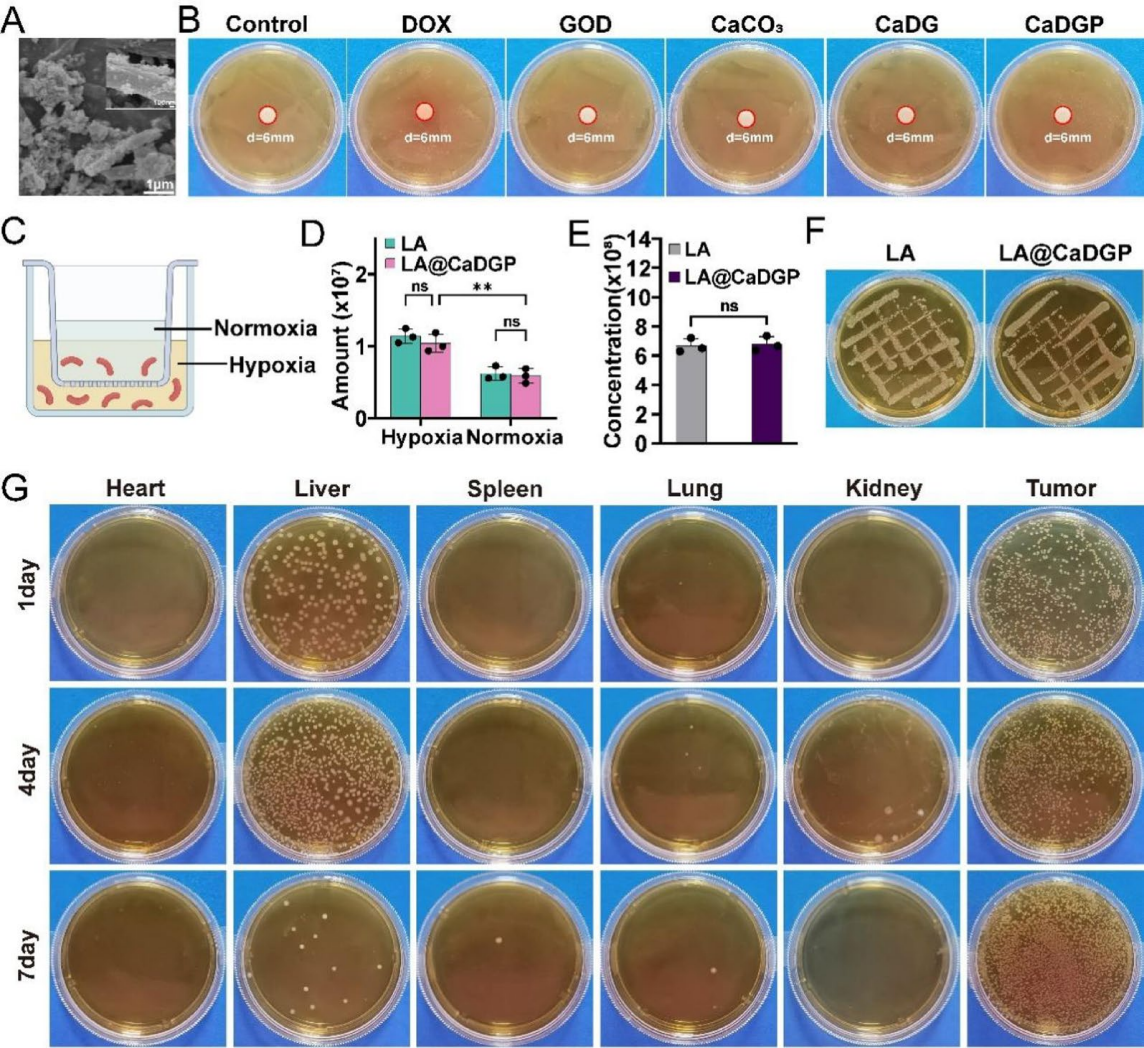
In vitro bioactivity evaluation and bacteria biodistribution in vivo. (**A**) SEM images of LA@CaDGP. scale bar: 1 μm, in inserted image, scale bar: 100 nm. (**B**) Antimicrobial activity of various drugs on LA. (**C**) In vitro hypoxia model simulated by Transwell. (**D**) Bacterial counts in Transwell chambers (*n* = 3). (**E-F**) Photographs and bacterial counts of LA and LA@CaDGP incubated anaerobically for 48 h (*n* = 3). (**G**) Representative pictures of bacteria growth in the heart, liver, spleen, lung, kidney, and tumor tissues of mice after oral administration of the LA@CaDGP biohybrids on days 1, 4, and 7. Results are presented as means ± SD. ns: no statistical significance, **P < 0.01

Due to the strong adhesive properties of polydopamine, CaDGP NPs were successfully linked with Lactobacillus acidophilus (LA). In the SEM images (Fig. S21 and Fig. 5A), spherical CaDGP NPs can be seen attached to the surfaces of the bacteria, resulting in the formation of biomotor LA@CaDGP. No inhibition rings were observed in any of the formulations, indicating that the various drugs did not hinder the growth of LA (Fig. 5B). When LA and LA@CaDGP were cultured on media for 48 h, both groups showed similar patterns of bacterial growth (Fig. 5E), and there was no significant difference in the results of bacterial counting (Fig. 5F). The targeting ability of LA and LA@CaDGP was then tested using Transwell chambers (Fig. 5C). After 2 h of incubation, a higher number of viable bacteria were found under hypoxic conditions compared to the upper normoxic conditions (Fig. 5D). Furthermore, there was no significant difference in number of bacteria between the LA and LA@CaDGP groups (*P >* 0.05), suggesting that the attachment of CaDGP NPs was compatible with the physiological activity and targeting ability of LA.

### In vivo targeting ability of the LA@CaDGP biomotors

**Fig. 6:**
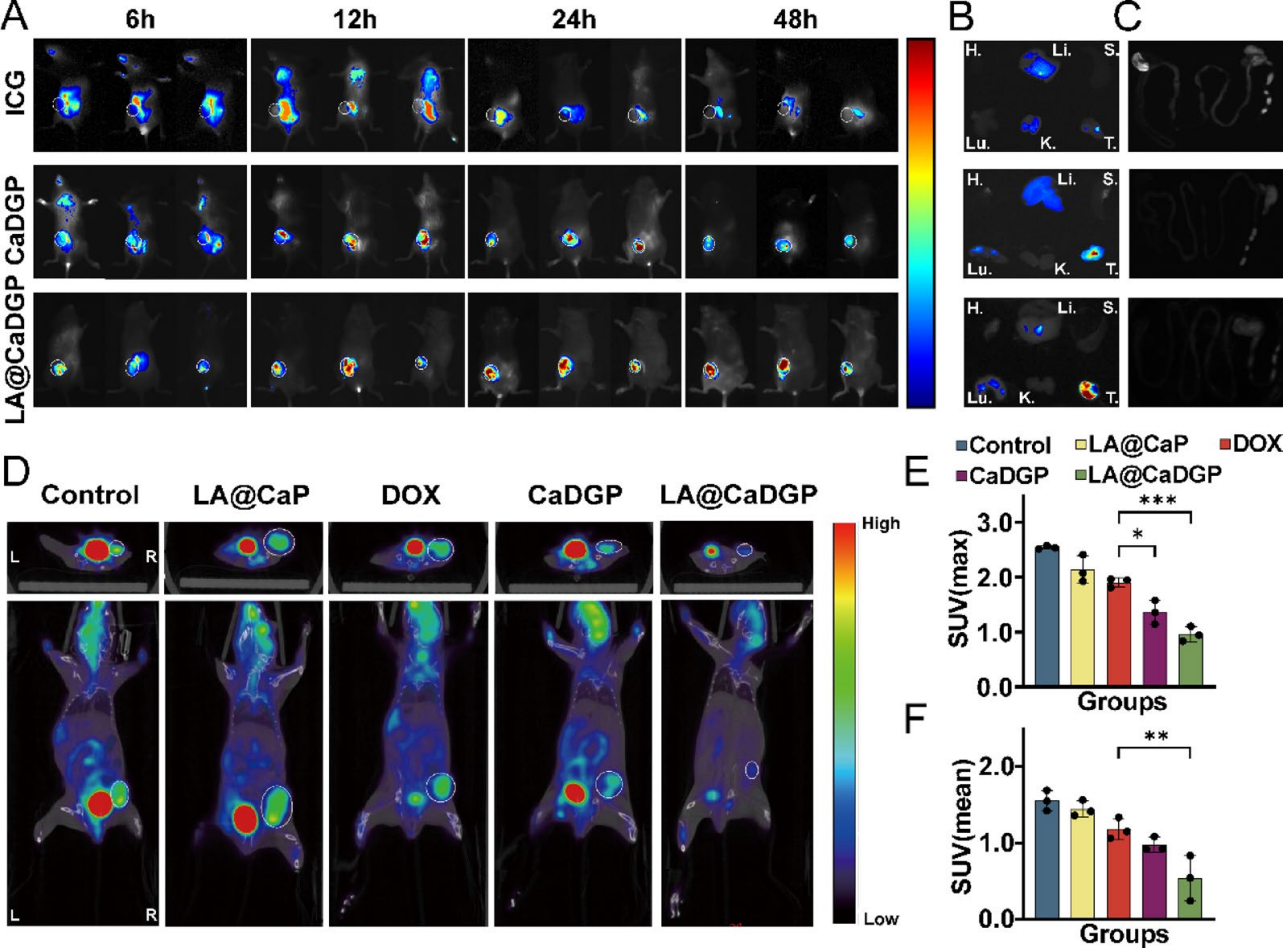
In vivo evaluation of drug biodistribution and early treatment response. (**A**) Fluorescence images of 4T1 tumor-bearing mice after oral administration of free ICG, ICG-loaded CaDGP, and ICG-loaded LA@CaDGP. (**B**) Fluorescence images of isolated organs and tumors at 48 h (H: heart, Li: liver, S: spleen, Lu: lung, K: kidney, T: tumor). (**C**) Fluorescence images of the harvested gastrointestinal tract at 48 h. (**D**) Representative images of ^18^F-FDGPET/CT on day 12 of treatment, upper: cross-sectional images, lower: coronal images. (**E**) SUVmax of each group (*n* = 3). (**F**) SUVmean of each group (*n* = 3). Results are presented as means ± SD. *P < 0.05, **P < 0.01, ***P < 0.001

The targeting ability of the LA@CaDGP biomotors was confirmed by evaluating the in vivo distribution of bacteria. After oral administration of the LA@CaDGP biomotors, the distribution of bacteria in the tumors and major organs of mice was assessed on days 1, 4, and 7. As shown in Fig. 5G and Fig. S22, bacteria primarily accumulated in the liver and tumors on days 1 and 4, indicating hepatic metabolism of the oral formulation. The number of bacteria in the major organs was significantly lower than in the tumor tissues, and by day 7, almost no bacteria were detected in other organs. As shown in Fig. 6A, the drug distribution of ICG-labeled CaDGP NPs and LA@CaDGP in mice was imaged at four time points following oral administration. In the LA@CaDGP group, the fluorescence signal quickly concentrated at the tumor site within 6 h and persisted for up to 48 h after administration, whereas the detectable fluorescence signals in the free ICG and ICG-labeled CaDGP groups gradually decreased. Ex vivo imaging of organs and tumor tissues (Fig. 6B and C) after 48 h revealed strong fluorescence signals in the tumor tissues of the LA@CaDGP group, while the free ICG and CaDGP groups showed weak signal retention. No significant fluorescence signal was observed in the gastrointestinal tract, indicating that the LA@CaDGP biomotors enhanced the accumulation and retention of therapeutic drugs in tumor tissues.

^18^F-FDG was injected into the mice in each group to assess early treatment response using PET/CT imaging. Figure 6D shows that the mice in the LA@CaDGP group exhibited the weakest metabolic signals in their tumors. Quantitative analysis of the maximum standardized uptake value (SUVmax, Fig. 6E) and mean SUV (SUVmean, Fig. 6F) confirmed that tumors in the LA@CaDGP group had the lowest uptake values, indicating that treatment with LA@CaDGP biomotors effectively inhibited glucose metabolism in tumor tissue.

### In vivo antitumoral effects

**Fig. 7:**
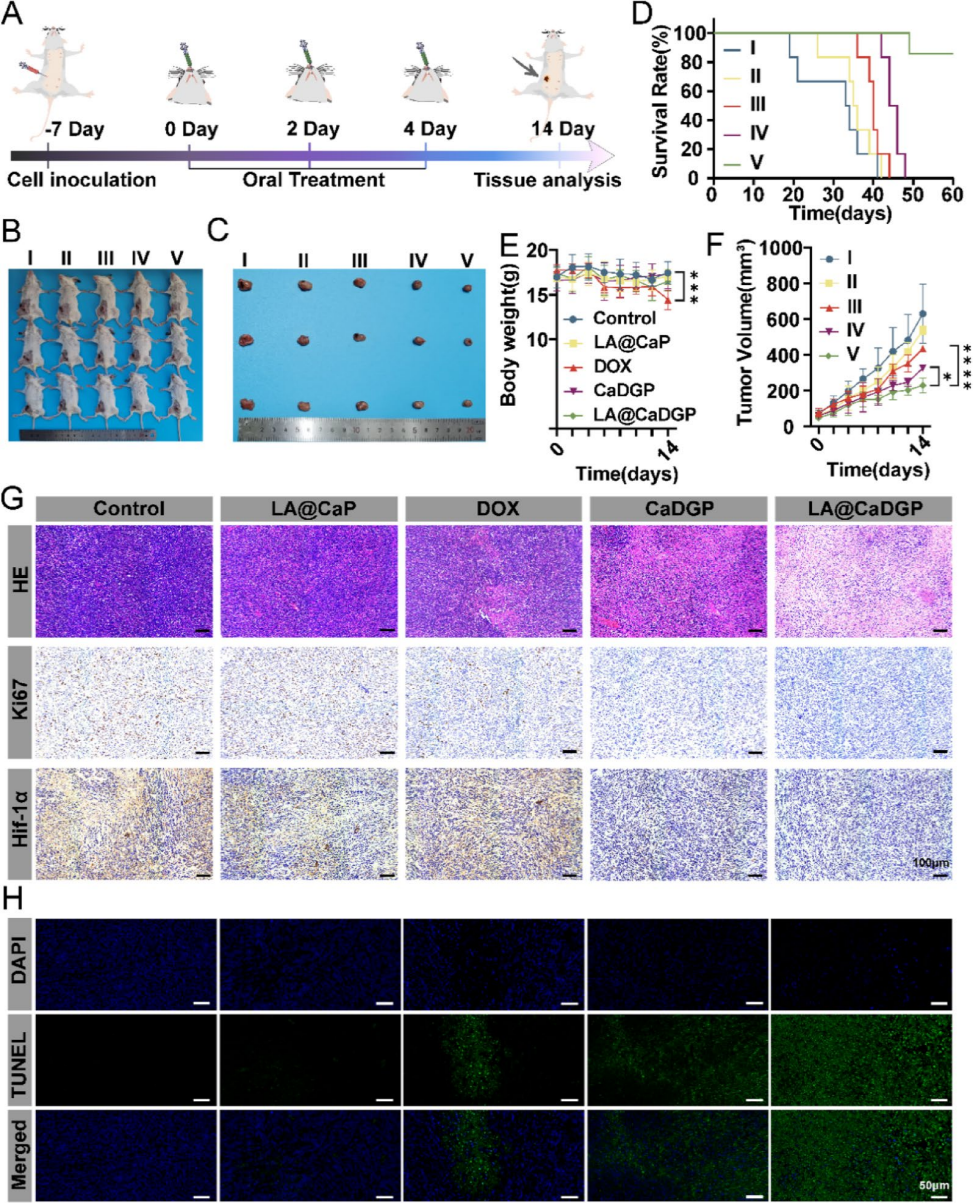
In vivo evaluation of anti-tumor effects. (**A**) Schematic diagram of the treatment program. (**B**) Representative photographs of 4T1 tumor-bearing mice on day 14 after treatment. (**C**) Representative photographs of isolated tumors.** (D**) Survival curves of mice (*n* = 6). (**E**) Body weight fluctuation during treatment (*n* = 6). (**F**) Curves of tumor volume (*n* = 6). (**G**) H&E, Ki67, Hif-1α staining, scale bar: 100 μm. (**H**) TUNEL staining of tumor tissues of mice, scale bar: 50 μm. Data are presented as means ± SD. *P < 0.05, ***P < 0.001, ****P < 0.0001. (group I: Control, group II: LA@CaP, group III: DOX, group IV: CaDGP, group V: LA@CaDGP)

According to the protocol shown in Fig. 7A, the anti-tumor efficacy of LA@CaDGP was evaluated using a 4T1 orthotopic tumor mouse model. On day 14 after treatment, the LA@CaDGP group exhibited the smallest tumor volume (229.77 ± 41.14 mm³) (Fig. 7B-C). The tumor growth curves shown in Fig. 7F indicate that the LA@CaDGP group experienced significantly inhibited tumor growth compared to the free DOX and CaDGP groups. Additionally, there was no significant difference in body weight between the LA@CaDGP and control groups throughout the entire treatment period, suggesting that LA@CaDGP treatment resulted in lower systemic toxicity (Fig. 7E). In contrast, the free DOX group experienced a substantial decrease in body weight after 14 days of treatment, likely due to toxicity resulting from poor targeting ability. Survival analysis (Fig. 7D) indicated that the median survival duration in the LA@CaDGP group exceeded 60 days, compared to approximately 45 days in the CaDGP group.

As illustrated in Fig. 7G, the results of H&E staining indicate extensive tumor cell necrosis in the LA@CaDGP group, characterized by widespread nuclear fragmentation and eosinophilic cytoplasmic changes (pink discoloration). The Ki67 images demonstrate the strong anti-proliferative effect of the LA@CaDGP biomotors. Furthermore, HIF-1α staining shows that LA@CaDGP treatment significantly reduced HIF-1α expression in tumor tissues, suggesting that the treatment greatly relieved tumor hypoxia. As shown in Fig. S23, compared with the control group, the percentage of Ki67-positive cells in the LA@CaDGP group decreased by approximately 99.5%, while the positive rate of HIF-1α was reduced to just one twentieth of that in the control group. In addition, the fluorescent images of TUNEL staining (Fig. 7H) reveal that tumor tissues in the LA@CaDGP group exhibited stronger green fluorescence, indicating enhanced apoptosis, which is further confirmed by the quantitative results (Fig. S23).

### Biosafety analysis of the LA@CaDGP biomotors

The biocompatibility of the LA@CaDGP biomotors was assessed using an in vitro hemolysis assay (Fig. S24). The results showed that the positive control (distilled water) caused complete lysis of erythrocytes, resulting in a clear red solution due to the release of hemoglobin. In contrast, all drug-treated groups exhibited negligible hemolysis, with erythrocyte morphology preserved under light microscopy and the solution remaining similar in appearance to the negative control (normal saline, NS). The hemolysis rate was determined by spectrophotometric quantification of the supernatants, based on the hemoglobin absorption peak at 540 nm. Even at a concentration of 2 mg/mL, the hemolysis rate of the LA@CaDGP biomotor remained below 5%, demonstrating its excellent blood biocompatibility.

**Fig. 8:**
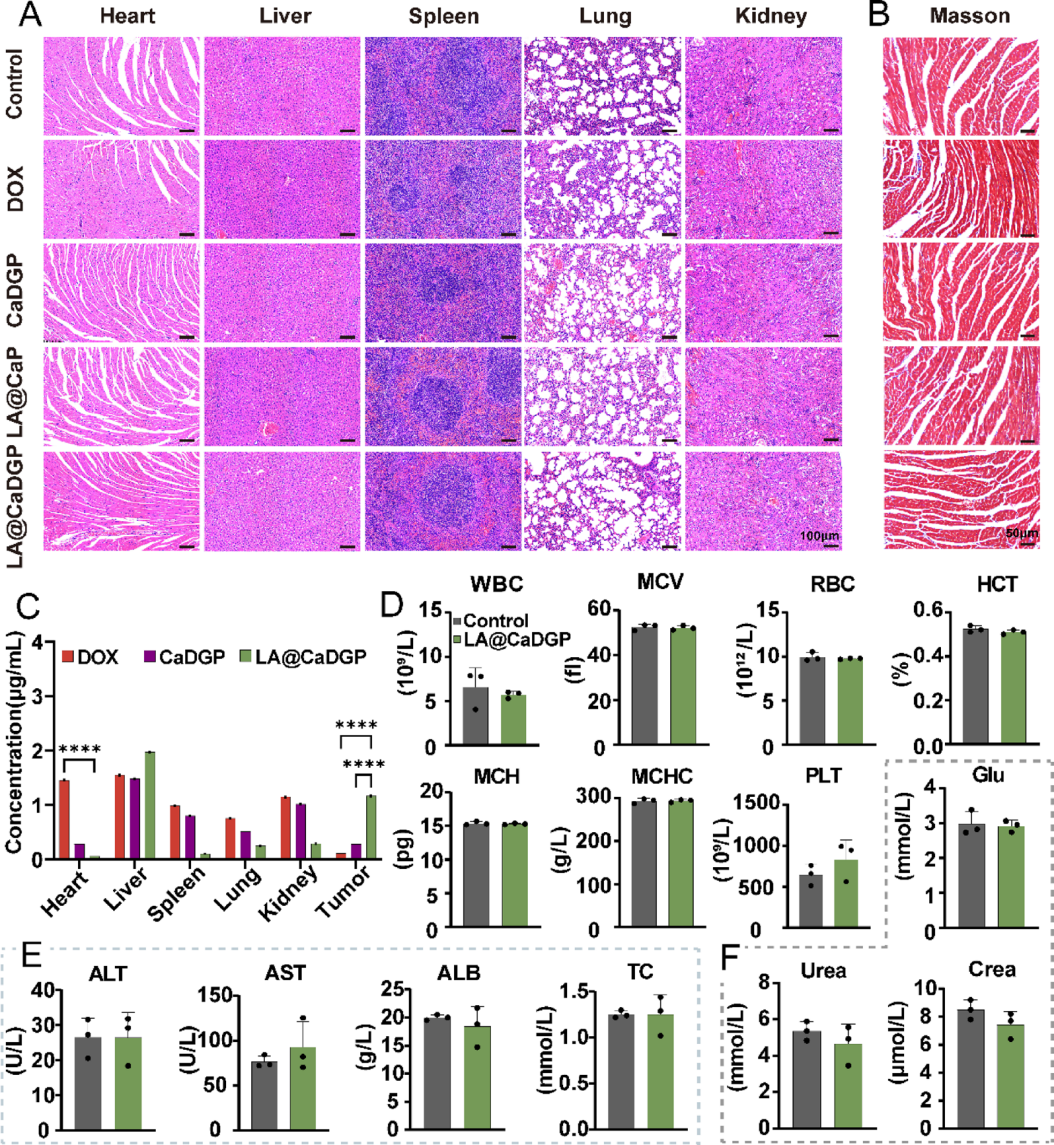
In vivo evaluation of biological safety and drug biodistribution. (**A**) H&E images of major organs (heart, liver, spleen, lung, and kidney) of mice, scale bar: 100 μm. (**B**) Masson staining of hearts in each group, scale bar: 50 μm. (**C**) DOX concentrations in major organs and tumors (*n* = 3). (**D**) Blood indices, biochemical markers of liver function (**E**) and (**F**) renal function (*n* = 3). Data are presented as means ± SD. ****P < 0.0001

Figure 8A shows images of H&E-stained isolated organs, revealing no significant abnormalities in the major organs. This suggests that treatment with LA@CaDGP results in reduced organ toxicity. Masson staining of heart tissues (Fig. 8B) also shows no prominent fibrosis in the myocardium of the LA@CaDGP group. Quantitative analysis of DOX concentrations in major organs and tumors (Fig. 8C) revealed substantial DOX accumulation in the liver, reflecting the hepatic metabolism of oral formulations. The LA@CaDGP group had a higher DOX concentration in tumor tissue (1.17 µg/mL), representing a 10.64-fold increase compared with the free DOX group and a 4.18-fold increase compared with the CaDGP group. Moreover, the DOX concentration in the heart of the LA@CaDGP group was significantly lower than that of the free DOX group, indicating that LA@CaDGP treatment not only enhanced drug accumulation at the tumor site but also effectively reduced DOX-induced cardiotoxicity. Results of routine blood tests and biochemical assays (Fig. 8D-F) showed no significant differences in various blood parameters between the LA@CaDGP group and the control group, further demonstrating the superior biosafety of the LA@CaDGP biomotors.

## Discussion

Although chemotherapy remains a cornerstone of breast cancer treatment, the associated systemic toxicity and adverse effects of chemotherapeutic agents continue to pose significant clinical challenges [41]. These shortcomings primarily arise from the non-targeted biodistribution of free chemotherapeutic drugs in vivo and the inherent limitations of monotherapy regimens [42]. The TME, which directly influences tumor growth and metastasis, represents a critical target for enhancing antitumor therapy [43, 4]. However, conventional chemotherapy formulations often fail to achieve sufficient drug accumulation within the TME, thereby compromising their anti-tumor efficacy. To address these challenges, we developed a bacteria-mediated nanoplatform utilizing hollow mesoporous calcium carbonate nanoparticles (CaCO_3_ NPs) for the dual delivery of glucose oxidase (GOD) and doxorubicin (DOX) to hypoxic tumor tissue. This approach enables synergistic glucose-deprivation (starvation) therapy and chemotherapy for the treatment of breast cancer.

Compared to passive targeting based on the enhanced permeability and retention (EPR) effect, actively targeted drug delivery systems can transport therapeutic agents into tumor cores with greater specificity and efficiency [44]. Currently, active targeting strategies primarily involve modifying the surface of nanoparticles with specific targeting molecules [45]. This approach facilitates drug delivery by allowing the targeting molecules to bind to designated receptors, antigens, or transporters on the surfaces of cells [46]. In reviewing the literature, we have has been particularly interested in the use of bacterial therapies for tumor treatment [47, 48]. Certain bacteria are not only naturally present in the digestive tract and within solid tumors [49, 50], but they are also able to specifically target and colonize tumor tissues due to their unique structural features and growth characteristics [51, 52]. In contrast, passive targeting relies on the abnormal permeability of tumor vasculature, making drug delivery susceptible to limitations imposed by hemodynamics and tumor heterogeneity [53]. This results in inefficient and unstable accumulation of drugs at tumor sites [54]. Compared to other active targeting strategies, such as antibody- or ligand-mediated molecular targeting, molecularly targeted drugs often cause off-target toxicity due to heterogeneous target expression and non-specific binding [55, 56]. Bacterial targeting, however, avoids common off-target effects and immunogenicity issues. Among these strategies, the unique characteristics of the TME—including hypoxia, hypernutrition, and immunosuppression—provide an ideal environment for the colonization of anaerobic bacteria such as Lactobacillus acidophilus (LA) [57]. These bacteria can migrate to tumor sites via chemotaxis, leveraging their metabolic advantages to survive within tumor tissues and release anti-tumor agents that directly target tumor cells and their microenvironment [58]. Simultaneously, the bacteria activate the host immune system, inducing immune cell infiltration and inflammation, thereby enhancing antitumor immunity and achieving multiple synergistic therapeutic effects [59]. In our previous work, targeted therapy for hypoxic lung tumors was achieved through intravenous administration of DOX-loaded nanomedicine using bacteria as drug carriers [60]. Given the superior patient compliance and dosing convenience associated with oral medications in clinical practice, we further investigated the feasibility of orally delivering bacteria-vectored DOX for targeted therapy of breast cancer [61]. In this study, we integrated starvation therapy with chemotherapy to amplify the therapeutic outcomes of bacteria-mediated oral delivery against breast tumors. Glucose oxidase (GOD) oxidizes interatumoral glucose, depleting nutrients essential for tumor survival and thus synergistically enhancing chemotherapeutic effects [62]. CaCO_3_ NPs neutralize tumor acidity and induce calcium overload through releasing Ca²⁺ ions, thereby triggering organelle damage. Compared to toxin-producing bacteria (e.g., Salmonella) or genetically engineered strains [63], probiotic Lactobacillus acidophilus (LA), a natural resident of the human gastrointestinal tract, was strategically selected in this study due to its demonstrated advantages in biosafety, clinical accessibility, and cost-effectiveness [64]. Subsequently, polydopamine (PDA) modification was employed to construct LA-based Biomotors, termed LA@CaDGP. This approach exploited PDA’s robust adhesiveness to firmly anchor nanodrugs onto the LA surface while simultaneously forming a protective coating on CaCO_3_ NPs [65]. This design prevents premature disintegration of the nanocarrier and leakage or degradation of the drugs. As demonstrated by Zeng et al. [66], PDA encapsulation significantly improved oral bioavailability. Consequently, the developed LA@CaDGP biomotors not only maintain bacterial bioactivity, but also efficiently deliver both DOX and GOD to tumor sites—a combination that is crucial for enhancing tumoricidal activity and minimizing systemic toxicity.

The in vitro and in vivo experimental data conclusively demonstrate that LA@CaDGP exhibits excellent biocompatibility and targeting specificity. Our findings show that bacterial growth did not differ significantly between the LA and LA@CaDGP groups after 48 h of incubation, indicating that the construction of biohybrids is compatible with the biological activity of the bacteria. This result is consistent with the research of Yang et al. [29]. In vivo biodistribution studies demonstrated that both LA and DOX significantly accumulated in the excised tumors of the LA@CaDGP group compared to other treatments, while cardiac DOX retention was reduced. This suggests that our strategy not only maintains the effective concentration of the drug in tumor tissues but also reduces the toxicity of DOX to major organs. Mice treated with LA@CaDGP biomotors exhibited the greatest survival advantage and inhibition of tumor progression. However, in the in vitro anaerobic propensity test, we observed slightly lower accumulation of LA in the anoxic chamber compared to the obligate anaerobic bacteria (Bif) reported previously [60], likely because LA belonging to facultative anaerobes. In addition, while LA@CaDGP showed significant bacterial accumulation in tumor tissue, high liver distribution was also observed in the short term. In future studies, we will optimize the composition and function of the LA@CaDGP biomotors to reduce bacterial distribution in normal organs. Furthermore, the anti-tumor efficacy of this bacteria-mediated drug delivery strategy should be validated in additional solid tumor models.

## Conclusion

In this study, a biologically driven, multilevel therapeutic nanomedicine named LA@CaDGP was designed to enable integrated chemotherapy and starvation therapy for breast cancer. Experimental results indicate that Lactobacillus acidophilus enhances the targeting ability of the drug to the tumor site, resulting in increased local concentrations of DOX and depletion of glucose. By altering the TME, this approach disrupts tumor cell metabolism through Ca^2+^ overload, causing damage to mitochondria and lysosomes, and works synergistically with DOX to induce cell death. In an in situ breast cancer mouse model, tumor growth was significantly inhibited and the survival time of the mice was notably extended. However, further validation is needed to confirm the anti-tumor efficacy in other solid tumors. Overall, the LA@CaDGP biomotor demonstrates excellent anti-tumor performance and good biosafety, offering a promising bacteria-based strategy for clinical applications in cancer therapy.

## Experimental section

### Materials and methods

Anhydrous calcium chloride (CAS:10043-52-4), Adriamycin hydrochloride, and glucose oxidase (100 U/mg) were sourced from Macklin Co., Ltd. (Shanghai, China). Lac. Acidophilus (BNCC336636; LOT: 240415) was sourced from the Henan Engineering Research Center of Industrial Microbiology (Henan, China). Tumor cell lines, including A549 (lung cancer), 4T1 (breast cancer), and CT26 (colorectal cancer), were procured from the Cell Bank of the Chinese Academy of Sciences (Shanghai, China). BALB/c female mice, obtained from Tengxin Experimental Animal Technology Co. (Chongqing, China), were housed under specific pathogen-free SPF conditions at Southwest Medical University. The animal facility was maintained at a constant ambient temperature of 24 °C and a relative humidity of 50%−60%, with a standardized 12-hour light/dark cycle. All mice were provided with a standard laboratory diet and filtered tap water ad libitum. The experimental protocols were reviewed and approved by the Institutional Animal Care and Use Committee of Southwest Medical University (Ethics Approval No.:20230828 − 012), in strict accordance with the National Research Council’s Guide for the Care and Use of Laboratory Animals. Throughout the study, no signs of infection or health abnormalities were observed in any of the mice.

### Preparation and characterization of CaCO_3_ NPs and CaDG NPs

A straightforward one-pot gas diffusion method was used to synthesize DOX-loaded CaCO_3_ nanoparticles (CaD NPs). Calcium chloride (CaCl_2_, 1 mM) was dissolved 50 mL of absolute ethanol in a beaker covered with aluminum foil. Separately, DOX (5 mg) was completely dissolved in 1 mL of absolute ethanol and added dropwise to the beaker. Small holes were punctured in the aluminum foil to allow gas exchange. The beaker was then placed in a vacuum-drying oven along with another beaker containing 2.5 g of dry ammonium bicarbonate (NH_4_HCO_3_). The reaction system was maintained under vacuum at 40℃ for 24 h. After the reaction, the resulting suspension was immediately centrifuged at 12,000 rpm for 15 min at 18℃. The precipitate was washed three times with ethanol to collect the CaD nanoparticles. Blank CaCO_3_ nanoparticles were prepared using the same method, but without the addition of DOX. To prepare GOD-loaded nanoparticles, GOD (5 mg) was pre-dissolved in 1 mL of demineralized water and then added dropwise to the CaD suspension, followed by stirring overnight. The GOD-loaded nanoparticles (CaDG NPs) were collected by centrifugation (12,000 rpm, 15 min) and washed with anhydrous ethanol. The morphology of the CaCO_3_ NPs and other synthetic products was observed using transmission electron microscopy (TEM, JEM1200EX, JEOL, Japan). Particle size and zeta potential were measured using dynamic light scattering (DLS, NanoBrook 90Puls Zeta, Brookhaven, NY).

### Preparation and characterization of CaDGP NPs

A suspension of CaDG NPs (0.5 mg/mL) was mixed with 150 µL of ammonia solution (1 M) and 1 mg of dopamine. After stirring for 12 h, the CaDGP NPs were collected and washed three times with anhydrous ethanol (12, 000 rpm, 15 min), and dried under vacuum at 37℃. The synthesis of CaDGP NPs was verified using energy dispersive spectroscopy (EDS), X-ray diffraction (XRD), X-ray photoelectron spectroscopy (XPS), and physical adsorption tests. The DOX content was determined by UV spectrophotometry (UV-5800, Metash Instruments, China). Drug loading (DL) and encapsulation efficiency (EE) were calculated using the following equations:

$$\:Loadingefficiency\:\left(\%\right)=\frac{\mathrm{W}\mathrm{L}\mathrm{o}\mathrm{a}\mathrm{d}\mathrm{e}\mathrm{d}\:\mathrm{D}\mathrm{O}\mathrm{X}}{\mathrm{W}\mathrm{C}aDGP}\times\:\:100\%$$

$$\begin{aligned}&\:Encapsulationefficiency\:\left(\%\right)\:\\& \quad=\frac{\mathrm{W}\mathrm{L}\mathrm{o}\mathrm{a}\mathrm{d}\mathrm{e}\mathrm{d}\:\mathrm{D}\mathrm{O}\mathrm{X}}{\mathrm{W}\mathrm{D}\mathrm{O}\mathrm{X}\:\mathrm{a}\mathrm{d}\mathrm{d}\mathrm{e}\mathrm{d}\:\mathrm{i}\mathrm{n}\mathrm{i}\mathrm{t}\mathrm{i}\mathrm{a}\mathrm{l}\mathrm{l}\mathrm{y}}\times\:\:100\%\end{aligned}$$

To determine the GOD content in CaDGP NPs, 1 mg of freeze-dried powder was pre-treated with 100 µL of 6 M HCl and dissolved in 1.9 mL of PBS (pH 7.4). The GOD content was assessed using a Bradford protein assay kit (Beyotime, China). To evaluate the catalytic activity of GOD, various samples (GOD, CaCO_3_, CaDG, and CaDGP) each containing an equal amount of glucose oxidase (100 µg/mL), were mixed with a glucose solution (1 mg/mL) at 37℃. The pH of the solution was monitored at different time intervals using a pH meter (METTLER TOLEDO, Shanghai, China).

### In vitro release of DOX

The kinetics of DOX release were investigated in PBS solutions. Samples containing a precise dosage of DOX were placed in PBS at varying pH levels (5.5, 6.5, and 7.4) and incubated at 37 °C with shaking. At predetermined time points, 2 mL of the supernatant was collected by centrifugation to measure the released DOX at 485 nm using a UV-Vis spectrophotometer (UV-5800, Metash Instruments, China). Subsequently, 2 mL of fresh PBS was added to the original release medium to maintain a constant volume for further testing. The cumulative release rate of DOX was quantified and graphically presented.

### In vitro cell migration assay and cellular uptake

The inhibitory effects of drug formulations on the migration of 4T1 and A549 cells were evaluated using an in vitro scratch assay. Cells were seeded in 6-well culture plates at a density of 5 × 10^5^ cells per well. Once the cells reached 95% confluence, a standardized linear scratch was created using 200-µL sterile pipette tips. After removing cellular debris removal by rinsing with PBS, the experimental groups were treated with drug-containing medium, each containing an equivalent dose of DOX (10 µM). Wound closure was assessed at 0, 12, and 24 h post-injury under phase-contrast microscopy (Olympus CKX53, Japan). Cell migration was quantified by measuring the reduction in wound area over time.

Tumor cells, including 4T1, CT26, and A549 were incubated in 6-well plates with DOX (5 µg/mL) in saline, DOX, CaDG NPs, and CaDGP NPs solutions for 4 h. After washing three times with PBS to remove unbound agents, the cells were counterstained with 4’,6-diamidino-2-phenylindole (DAPI) for 20 min. After three additional PBS washes, intracellular DOX levels were assessed using a fluorescence microscope (Olympus, BX53, Japan) and quantified by flow cytometry (BD, FACSAria, America).

### In vitro study of cell viability and bacteriostasis assay

4T1, A549, and CT26 tumor cells were seeded in 96-well plates and treated with various drug formulations (CaCO_3_, DOX, CaDG, and CaDGP) at pH 7.4 or pH 6.5, using a range of DOX concentrations (1.25–40 µg/mL) for 24 h. Subsequently, 20 µL of thiazolyl blue tetrazolium bromide (MTT) solution (5 mg/mL) was added to each well, followed by an additional 1-hour incubation. The resulting formazan crystals were dissolved in 150 µL of dimethyl sulfoxide, and the optical density was measured at 490 nm using a microplate reader (BIO-RAD, iMark, America).

A bacterial suspension of LA was uniformly inoculated onto semisolid agar plates. Sterile 6-mm antimicrobial disks were impregnated with solutions of DOX, GOD, CaCO_3_, CaDG, CaDGP, or saline. The disks were air-dried and aseptically transferred to the centers of the plates. Following incubation at 37 °C for 48 h, the zones of bacterial growth inhibition surrounding the disks were measured.

### In vitro evaluation of intracellular Ca^2+^ levels, lysosomal membrane integrity, and damage of mitochondrion

First, intracellular calcium deposits were visualized using Alizarin Red staining. Tumor cells, including 4T1, A549, and CT26, were cultured overnight and subsequently incubated with 2 mL of CaDGP solution. At designated time points (3, 6, 12, and 24 h), the cells were washed with PBS, fixed with was 95% ethanol, and then stained with 2 mL of 0.2% Alizarin Red solution (pH 8.3) for 30 min. Cellular morphology and calcium deposition were examined under a light microscope (Olympus CKX53, Japan). In parallel experiments, tumor cells were seeded into 6-well plates at a density of 2.0 × 10⁵ cells/well and cultured for 24 h. Subsequently, the cells were treated with different agents, including saline, CaCO_3_ NPs, CaDG NPs, CaDGP NPs, and CaDGP NPs. Specifically, in the CaDGP NPs group, BAPTA-AM, a Ca²⁺ chelator, was added. After 4 h of drug treatment for, the cells were washed and stained with Fluo-4 AM at 37℃ for 30 min, and then imaged using a fluorescence microscope (Olympus TX73, Japan).

For assessment of lysosomal membrane integrity, tumor cells were seeded in 6-well culture dishes at a density of 1.0 × 10⁶ cells/well and treated with either saline or CaDGP NPs for 24 h. Following staining with acridine orange (5 µM) for 15 min, the cells were washed twice with PBS and analyzed by fluorescence microscopic (Olympus IX73, Tokyo, Japan). To assess mitochondrial membrane potential (MMP), tumor cells were treated as described above. After removing the culture medium, JC-1 dye was added, and the cells were incubated for 30 min at 37℃, followed by three washes. MMP was then detected and analyzed using a fluorescence microscope (Olympus BX53, Japan).

### Intracellular hydroxyl radicals •OH generation

Tumor cells, including 4T1, A549, and CT26, were exposed to 0.1 mL of various test formulations: saline, DOX, GOD, CaCO_3_, CaDG, and CaDGP for 12 h. After washing three times with PBS, the cells were incubated with 0.2 mL of DCFH-DA (0.1 M) and 1.8 mL of PBS (pH 6.8) for 30 min. Intracellular DCF fluorescence (ex = 488 nm, em = 525 nm) was measured under an inverted fluorescence microscope (Olympus IX73, Japan) to quantify the production of •OH.

### Preparation and characterization of LA@CaDGP biomotor

A CaDGP solution (8 mL, 1 mg/mL) and an LA suspension (100 µL, 2.0 × 10^7^ CFU/mL) were mixed and incubated at 37 °C for 3 h. The mixture was then centrifuged at 2,700 rpm for 3 min and washed three times. The morphological characteristics of the LA@CaDGP biomotors were analyzed by scanning electron microscopy (SEM, Hitachi model SU 8020, Japan). To assess the antibacterial efficacy of CaDGP NPs, fresh LA@CaDGP NPs were cultured on agar plates. After 48 h of incubation, viable bacterial counts were determined. Naked LA was used as the control.

### In vitro targeting capability of the LA@CaDGP biomotor

To evaluate anaerobic bioactivity under simulated TME conditions, a hypoxia-mimicking Transwell system was established. In the upper chambers, 200 µL suspensions of LA and LA@CaDGP (3.0 × 10^8^ CFU/mL) were introduced, respectively. The lower chambers received 400 µL of an enzymatic hypoxia-inducing system containing catalase (0.5 KU), glucose solution (10 µM), and glucose oxidase (0.5 KU). After a 2-hour hypoxic incubation, viable bacteria that migrated to the lower chambers were counted.

### In vivo anti-tumor efficacy

To establish an in-situ breast tumor model, female Balb/c mice were subcutaneously inoculated with a suspension of 4T1 cells (100 µL, 5.0 × 10^6^ cells) into the fourth thoracic mammary gland. Tumor volumes were calculated using the formula: $\:Tumor\:volume=\frac{(\mathrm{L}\mathrm{e}\mathrm{n}\mathrm{g}\mathrm{t}\mathrm{h}\:\times\:\:\mathrm{W}\mathrm{i}\mathrm{d}{t}{h}^2)}{2}$. When tumors reached a volume of 50–80 mm^3^, the mice were randomized into five experimental groups, including I: saline (NS), II: DOX, III: CaDGP NPs, IV: LA@CaP NPs, and V: LA@CaDGP NPs. The corresponding treatments were administered via oral gavage on alternate days for a total of three administrations, maintaining a DOX dosage of 15 mg/kg. Body weight was recorded every two days throughout the 14-day study period. On day 14, primary organs (heart, liver, spleen, lung, and kidney) and tumor tissues were collected for histological analysis.

Early therapeutic responses to different treatments in tumor-bearing mice were assessed using microPET/CT (Siemens, Germany). Following a 6-hour fasting period, the mice received intravenous injections of 200–250 µCi of 18 F-FDG. After a one-hour tracer uptake period, anesthesia was induced with isoflurane, and whole-body PET/CT scans were acquired in 2D mode, with an emission scan lasting 10 min per position. The scans were performed under the following parameters: tube voltage of 80 kV, tube current of 500 mA, and a collimated slice thickness of 1.5 mm. Image analysis was conducted by two board-certified nuclear medicine radiologists who were blinded to the treatment groups. Tumor volumes were delineated by manually drawing volumetric regions of interest, and quantitative PET metrics were calculated as the maximum standardized uptake value (SUVmax) and the mean uptake value (SUVmean).

### In vivo targeting ability and biodistribution of LA@CaDGP

The mice were orally administered 200 µL of LA@CaDGP once their tumors reached a volume of to 50 mm³. At designated time points (days 1, 3, and 7 post-treatment), primary organ specimens (cardiac, hepatic, splenic, pulmonary, and renal tissues), along with tumors, were surgically excised. Tissue processing began immediately: the samples were ground, homogenized, and plated onto Petri dishes. Bacterial colonization was assessed after incubating the plates for 48 h under anaerobic conditions at 37℃.

In addition, the tumor-bearing mice received various DOX-loaded drugs at an equal dosage (10 mg/kg). Twenty-four hours later, the organs were aseptically collected, weighed, and mechanically homogenized. The homogenate from each tissue was mixed with 3 mL of precipitant reagent, vortexed vigorously for 30 s, and centrifuged at 4℃ (10,000 rpm, 10 min). The supernatant was collected for analysis. DOX quantification was performed using a fluorescence spectrophotometer (FS5, Edinburgh Instruments, UK) with excitation/emission wavelengths of 480 ± 5 nm and 570 ± 10 nm, respectively.

### In vivo fluorescence imaging

ICG-conjugated LA@CaDGP NPs were synthesized by incubating 5 mg of ICG with 5 mL of LA@CaDGP NPs suspension (1 mg/mL) for 24 h at 25 °C in the dark. Unbound ICG was removed by centrifuging the mixture three times, followed by washing with PBS. When the tumor volume reached approximately 200 mm³, mice were orally administered 0.1 mL of either ICG, CaDGP-NPs/ICG, or LA@CaDGP-NPs/ICG, with each group receiving an equivalent ICG dose (1 mg/kg). Images were acquired at 6, 12, 24, and 48 h post administration using the IVIS imaging system (Series III 900/1700, Suzhou, China). After 48 h, tumor tissues, major organs, and gastrointestinal tracts from each group were imaged using the same platform.

### In vitro hemolysis assay and in vivo hematological analysis

During hemocompatibility assessments, 1 mL aliquots of 0.2% v/v erythrocyte suspensions were co-incubated with 1 mL of saline solutions containing LA@CaDGP at concentrations ranging from 0.25 to 2.0 mg/mL, while maintaining at 37 °C for 4 h. Red blood cells incubated with hypotonic solution (ddH_2_O) and saline served as positive and negative controls, respectively. Following centrifugation at 3,000 rpm for 10 min, the optical density (OD) of each supernatant was measured at 540 nm and the hemolysis rates were calculated using the following formula:

$$\begin{aligned}&\:\mathrm{H}\mathrm{e}\mathrm{m}\mathrm{o}\mathrm{l}\mathrm{y}\mathrm{s}\mathrm{i}\mathrm{s}\:\mathrm{r}\mathrm{a}\mathrm{t}\mathrm{e}\:\left({\%}\right) \\&\quad=\frac{(\mathrm{O}\mathrm{D}\:\mathrm{v}\mathrm{a}\mathrm{l}\mathrm{u}\mathrm{e}\:\mathrm{o}\mathrm{f}\:\mathrm{t}\mathrm{h}\mathrm{e}\:\mathrm{e}\mathrm{x}\mathrm{p}\mathrm{e}\mathrm{r}\mathrm{i}\mathrm{m}\mathrm{e}\mathrm{n}\mathrm{t}\mathrm{a}\mathrm{l}\:\mathrm{g}\mathrm{r}\mathrm{o}\mathrm{u}\mathrm{p}-\mathrm{O}\mathrm{D}\:\mathrm{v}\mathrm{a}\mathrm{l}\mathrm{u}\mathrm{e}\:\mathrm{o}\mathrm{f}\:\mathrm{t}\mathrm{h}\mathrm{e}\:\mathrm{s}\mathrm{a}\mathrm{l}\mathrm{i}\mathrm{n}\mathrm{e}\:\mathrm{g}\mathrm{r}\mathrm{o}\mathrm{u}\mathrm{p})}{(\mathrm{O}\mathrm{D}\:\mathrm{v}\mathrm{a}\mathrm{l}\mathrm{u}\mathrm{e}\:\mathrm{o}\mathrm{f}\:\mathrm{t}\mathrm{h}\mathrm{e}\:\mathrm{p}\mathrm{o}\mathrm{s}\mathrm{i}\mathrm{t}\mathrm{i}\mathrm{v}\mathrm{e}\:\mathrm{c}\mathrm{o}\mathrm{n}\mathrm{t}\mathrm{r}\mathrm{o}\mathrm{l}\:\mathrm{g}\mathrm{r}\mathrm{o}\mathrm{u}\mathrm{p}-\mathrm{O}\mathrm{D}\:\mathrm{v}\mathrm{a}\mathrm{l}\mathrm{u}\mathrm{e}\:\mathrm{o}\mathrm{f}\:\mathrm{t}\mathrm{h}\mathrm{e}\:\mathrm{s}\mathrm{a}\mathrm{l}\mathrm{i}\mathrm{n}\mathrm{e}\:\mathrm{g}\mathrm{r}\mathrm{o}\mathrm{u}\mathrm{p})} \\&\quad\times\:100\%\end{aligned}$$

To evaluate in vivo biosafety, healthy Kunming mice were orally administered LA@CaDGP NPs at a dose of 15 mg/kg DOX on days 1, 3, and 5. Control mice received isovolumetric doses of physiological saline. Blood samples were collected at the experimental endpoint (day 14 post-initiation) via retro-orbital venous plexus puncture under isoflurane anesthesia. Routine hematological and biochemical parameters including RBC, HCT, MCHC, MCH, MCV, WBC, PLT, ALT, AST, ALB, TC, urea, Crea, and Glu were measured.

### Statistical analysis

Quantitative results are presented as the mean ± standard deviation (SD). Statistical comparisons between two groups were performed using Student’s t-test, while comparisons among multiple groups were conducted using one-way ANOVA. Statistical significance was defined as *P* < 0.05.

## Supplementary Information

Supplementary Material 1

## Data Availability

All data needed to support the conclusions are present in the paper and/or the Supplementary Materials. Additional data related to this paper may be requested from the authors.

## References

[1] Xiong X, Zheng LW, Ding Y, Chen YF, Cai YW, Wang LP, et al. Breast cancer: pathogenesis and treatments. Sig Transduct Target Ther. 2025;10:49. 10.1038/s41392-024-02108-4 10.1038/s41392-024-02108-4 PMC11836418 39966355

[2] Loibl S, Poortmans P, Morrow M, Denkert C, Curigliano G. Breast cancer. Lancet. 2021;397:1750–69. 10.1016/S0140-6736(20)32381-3. 33812473 10.1016/S0140-6736(20)32381-3

[3] Zhang J. Chemotherapy-elicited exosomal miR-378a-3p and miR-378d to promote breast cancer stemness and chemoresistance via the activation of EZH2/STAT3 signaling. J Clin Oncol. 2021;39:e12615. 10.1200/JCO.2021.39.15_suppl.e12615 10.1186/s13046-021-01901-1 PMC8022546 33823894

[4] Harris MA, Savas P, Virassamy B, O’Malley MMR, Kay J, Mueller SN, et al. Towards targeting the breast cancer immune microenvironment. Nat Rev Cancer. 2024;24:554–77. 10.1038/s41568-024-00714-6. 38969810 10.1038/s41568-024-00714-6

[5] Francis A, Venkatesh GH, Zaarour RF, Zeinelabdin NA, Nawafleh HH, Prasad P, et al. Tumor hypoxia: a key determinant of microenvironment hostility and a major checkpoint during the antitumor response. Crit Rev Immunol. 2018;38:505–24. 10.1615/CritRevImmunol.2019030168. 31002604 10.1615/CritRevImmunol.2019030168

[6] Kalluri R. The biology and function of fibroblasts in cancer. Nat Rev Cancer. 2016;16:582–98. 10.1038/nrc.2016.73. 27550820 10.1038/nrc.2016.73

[7] de Melo MDT, Almeida ALC, Neto AJ, de OM, Júnior EG, dos S, Reis IC, Mota L, et al. Which therapeutic strategy is less cardiotoxic in the treatment of breast cancer: using Paclitaxel before or after doxorubicin? JCO. 2021;39:e12617. 10.1200/JCO.2021.39.15_suppl.e12617

[8] Amaro-Leal Â, Machado F, Afonso AI, Rocha I, Geraldes V. Autonomic and cardiac evaluation upon sub-therapeutic doxorubicin administration. Eur Heart J. 2021;42:ehab724.3332. 10.1093/eurheartj/ehab724.3332.

[9] Tarantino P, Carmagnani Pestana R, Corti C, Modi S, Bardia A, Tolaney SM, et al. Antibody–drug conjugates: smart chemotherapy delivery across tumor histologies. CA Cancer J Clin. 2022;72:165–82. 10.3322/caac.21705. 34767258 10.3322/caac.21705

[10] Ijäs H, Shen B, Heuer-Jungemann A, Keller A, Kostiainen MA, Liedl T, et al. Unraveling the interaction between doxorubicin and DNA origami nanostructures for customizable chemotherapeutic drug release. Nucleic Acids Res. 2021;49:3048–62. 10.1093/nar/gkab097. 33660776 10.1093/nar/gkab097 PMC8034656

[11] Cortes J, Rugo HS, Cescon DW, Im S-A, Yusof MM, Gallardo C, et al. Pembrolizumab plus chemotherapy in advanced triple-negative breast cancer. N Engl J Med. 2022;387:217–26. 10.1056/NEJMoa2202809. 35857659 10.1056/NEJMoa2202809

[12] Kude de Almeida F, Soares Falcetta F, Dornelles Rosa D. Benefits and risks of anthracyclines in early-stage breast cancer. Lancet. 2024;403:1239–40. 10.1016/S0140-6736(23)02889-1. 38555132 10.1016/S0140-6736(23)02889-1

[13] Lu R, Wang S, Yang Z, Zhou L, Yang C, Wu Y. Accurate programmed multifunctional nano-missiles for self-promoted deep delivery and synergistic cascade tumor therapy: tactfully collaborating chemosynthesis with tumor microenvironment remodeling. Theranostics. 2022;12:5299–316. 10.7150/thno.74550. 35910803 10.7150/thno.74550 PMC9330536

[14] Lee SY, Seo J, Kim S, Hwang C, Jeong DI, Park J, et al. Cuproptosis-inducible chemotherapeutic/cascade catalytic reactor system for combating with breast cancer. Small. 2023;19:2301402. 10.1002/smll.202301402. 10.1002/smll.202301402 37162448

[15] Pan Y, Zhu Y, Xu C, Pan C, Shi Y, Zou J, et al. Biomimetic yolk–shell nanocatalysts for activatable dual-modal-image-guided triple-augmented chemodynamic therapy of cancer. ACS Nano. 2022;16:19038–52. 10.1021/acsnano.2c08077. 36315056 10.1021/acsnano.2c08077

[16] Huang H, Zhang C, Wang X, Shao J, Chen C, Li H, et al. Overcoming hypoxia-restrained radiotherapy using an erythrocyte-inspired and glucose-activatable platform. Nano Lett. 2020;20:4211–9. 10.1021/acs.nanolett.0c00650. 32352796 10.1021/acs.nanolett.0c00650

[17] Jo SM, Wurm FR, Landfester K. Oncolytic nanoreactors producing hydrogen peroxide for oxidative cancer therapy. Nano Lett. 2020;20:526–33. 10.1021/acs.nanolett.9b04263 31789526 10.1021/acs.nanolett.9b04263

[18] Zhang Y, Jiang S, Lin J, Huang P. Antineoplastic enzyme as drug carrier with activatable catalytic activity for efficient combined therapy. Angew Chem Int Ed Engl. 2022;61:e202208583. 10.1002/anie.202208583. 35848681 10.1002/anie.202208583

[19] Pan L, Liu J, Shi J. Cancer cell nucleus-targeting nanocomposites for advanced tumor therapeutics. Chem Soc Rev. 2018;47:6930–46. 10.1039/c8cs00081f. 30062349 10.1039/c8cs00081f

[20] Liu Y, Wang Y, Song S, Zhang H. Tumor diagnosis and therapy mediated by metal phosphorus-based nanomaterials. Adv Mater. 2021;33:e2103936. 10.1002/adma.202103936. 34596931 10.1002/adma.202103936

[21] Sharma P, Otto M. Multifunctional nanocomposites modulating the tumor microenvironment for enhanced cancer immunotherapy. Bioact Mater. 2024;31:440–62. 10.1016/j.bioactmat.2023.08.022. 37701452 10.1016/j.bioactmat.2023.08.022 PMC10494322

[22] Dykman L, Khlebtsov B, Khlebtsov N. Drug delivery using gold nanoparticles. Adv Drug Deliv Rev. 2025;216:115481. 10.1016/j.addr.2024.115481. 39617254 10.1016/j.addr.2024.115481

[23] Chang M, Hou Z, Jin D, Zhou J, Wang M, Wang M, et al. Colorectal tumor microenvironment-activated bio‐decomposable and metabolizable Cu_2_O@CaCO_3_ nanocomposites for synergistic oncotherapy. Adv Mater. 2020;32:2004647. 10.1002/adma.202004647 10.1002/adma.202004647 32945002

[24] Zheng P, Ding B, Shi R, Jiang Z, Xu W, Li G, et al. A multichannel Ca^2+^ nanomodulator for multilevel mitochondrial destruction-mediated cancer therapy. Adv Mater. 2021;33:2007426. 10.1002/adma.202007426 10.1002/adma.202007426 33675268

[25] Xu W, Suo A, Aldai AJM, Wang Y, Fan J, Xia Y, et al. Hollow calcium/copper bimetallic amplifier for Cuproptosis/Paraptosis/Apoptosis cancer therapy via cascade reinforcement of endoplasmic reticulum stress and mitochondrial dysfunction. ACS Nano. 2024;18:30053–68. 10.1021/acsnano.4c11455. 39412236 10.1021/acsnano.4c11455

[26] Liu J, Zhu C, Xu L, Wang D, Liu W, Zhang K, et al. Nanoenabled intracellular calcium bursting for safe and efficient reversal of drug resistance in tumor cells. Nano Lett. 2020;20:8102–11. 10.1021/acs.nanolett.0c03042. 33064007 10.1021/acs.nanolett.0c03042

[27] Dong M, Yang X, Zhang W, Qiu Y, Song P, Liu H, et al. Living therapeutics of nonpathogenic bacteria as biosynthesis factory and active carriers for enhancing tumor-targeted therapy. Nat Commun. 2025;16:6532. 10.1038/s41467-025-61675-4. 40664636 10.1038/s41467-025-61675-4 PMC12264139

[28] Yu Y, Wang Y, Zhang J, Bu Q, Jiang D, Jiang Y, et al. Anaerobic probiotics-in situ se nanoradiosensitizers selectively anchor to tumor with immuno-regulations for robust cancer radio-immunotherapy. Biomaterials. 2025;318:123117. 10.1016/j.biomaterials.2025.123117. 39864125 10.1016/j.biomaterials.2025.123117

[29] Yang Y, Hu T, Bian Y, Meng F, Yu S, Li H, et al. Coupling probiotics with 2D CoCuMo-LDH nanosheets as a tumor‐microenvironment‐responsive platform for precise NIR‐II photodynamic therapy. Adv Mater. 2023;35:e2211205. 10.1002/adma.202211205 10.1002/adma.202211205 36913539

[30] Liu H, Shen S, Xu Q, Wang Y, Qi K, Lu B, et al. Noncanonical amino acids as prophage inducers for protein regulation in bacteria-based delivery systems. mBio. 2025;16:e0398824. 10.1128/mbio.03988-24. 40084898 10.1128/mbio.03988-24 PMC11980383

[31] He J, Li C, Ding L, Huang Y, Yin X, Zhang J, et al. Tumor targeting strategies of smart fluorescent nanoparticles and their applications in cancer diagnosis and treatment. Adv Mater. 2019;31:1902409. 10.1002/adma.201902409. 10.1002/adma.201902409 31369176

[32] Hao J-N, Ge K, Chen G, Dai B, Li Y. Strategies to engineer various nanocarrier-based hybrid catalysts for enhanced chemodynamic cancer therapy. Chem Soc Rev. 2023;52:7707–36. 10.1039/D3CS00356F. 37874584 10.1039/d3cs00356f

[33] An P, Fan F, Gu D, Gao Z, Hossain AMS, Sun B. Photothermal-reinforced and glutathione-triggered in situ cascaded nanocatalytic therapy. J Control Release. 2020;321:734–43. 10.1016/j.jconrel.2020.03.007. 32145265 10.1016/j.jconrel.2020.03.007

[34] Ruan H, Hu Q, Wen D, Chen Q, Chen G, Lu Y, et al. A dual-bioresponsive drug-delivery depot for combination of epigenetic modulation and immune checkpoint blockade. Adv Mater. 2019;31:e1806957. 10.1002/adma.201806957. 30856290 10.1002/adma.201806957

[35] Fang T, Cao X, Wang L, Chen M, Deng Y, Chen G. Bioresponsive and immunotherapeutic nanomaterials to remodel tumor microenvironment for enhanced immune checkpoint blockade. Bioact Mater. 2024;32:530–42. 10.1016/j.bioactmat.2023.10.023. 38026439 10.1016/j.bioactmat.2023.10.023 PMC10660011

[36] Fu LH, Qi C, Lin J, Huang P. Catalytic chemistry of glucose oxidase in cancer diagnosis and treatment. Chem Soc Rev. 2018;47:6454–72. 10.1039/C7CS00891K 30024579 10.1039/c7cs00891k

[37] Delfino I, Portaccio M, Ventura BD, Mita DG, Lepore M. Enzyme distribution and secondary structure of sol–gel immobilized glucose oxidase by micro-attenuated total reflection FT-IR spectroscopy. Mater Sci Eng C Mater Biol. 2013;33:304–10. 10.1016/j.msec.2012.08.044. 10.1016/j.msec.2012.08.044 25428076

[38] Wu J, Cai X, Williams GR, Meng Z, Zou W, Yao L, et al. 2D antimonene-integrated composite nanomedicine for augmented low-temperature photonic tumor hyperthermia by reversing cell thermoresistance. Bioact Mater. 2022;10:295–305. 10.1016/j.bioactmat.2021.08.018. 34901547 10.1016/j.bioactmat.2021.08.018 PMC8636770

[39] Ding B, Shao S, Yu C, Teng B, Wang M, Cheng Z, et al. Large-pore mesoporous‐silica‐coated upconversion nanoparticles as multifunctional immunoadjuvants with ultrahigh photosensitizer and antigen loading efficiency for improved cancer photodynamic immunotherapy. Adv Mater. 2018;30:1802479. 10.1002/adma.201802479. 10.1002/adma.201802479 30387197

[40] Song Q, Zheng C, Jia J, Zhao H, Feng Q, Zhang H, et al. A probiotic spore-based oral autonomous nanoparticles generator for cancer therapy. Adv Mater. 2019;31:1903793. 10.1002/adma.201903793. 10.1002/adma.201903793 31490587

[41] Craig M, Jenner AL, Namgung B, Lee LP, Goldman A. Engineering in medicine to address the challenge of cancer drug resistance: from micro- and nanotechnologies to computational and mathematical modeling. Chem Rev. 2021;121:3352–89. 10.1021/acs.chemrev.0c00356. 33152247 10.1021/acs.chemrev.0c00356

[42] Nitheesh Y, Pradhan R, Hejmady S, Taliyan R, Singhvi G, Alexander A, et al. Surface engineered nanocarriers for the management of breast cancer. Mater Sci Eng C. 2021;130:112441. 10.1016/j.msec.2021.112441 10.1016/j.msec.2021.112441 34702526

[43] Zhou Q, Xiang J, Qiu N, Wang Y, Piao Y, Shao S, et al. Tumor abnormality-oriented nanomedicine design. Chem Rev. 2023;123:10920–89. 10.1021/acs.chemrev.3c00062. 37713432 10.1021/acs.chemrev.3c00062

[44] Izci M, Maksoudian C, Manshian BB, Soenen SJ. The use of alternative strategies for enhanced nanoparticle delivery to solid tumors. Chem Rev. 2021;121:1746–803. 10.1021/acs.chemrev.0c00779. 33445874 10.1021/acs.chemrev.0c00779 PMC7883342

[45] Vincent MP, Navidzadeh JO, Bobbala S, Scott EA. Leveraging self-assembled nanobiomaterials for improved cancer immunotherapy. Cancer Cell. 2022;40:255–76. 10.1016/j.ccell.2022.01.006. 35148814 10.1016/j.ccell.2022.01.006 PMC8930620

[46] Xiao Y, Yu D. Tumor microenvironment as a therapeutic target in cancer. Pharmacol Ther. 2021;221:107753. 10.1016/j.pharmthera.2020.107753. 33259885 10.1016/j.pharmthera.2020.107753 PMC8084948

[47] Zhu D, Pan W, Li H, Hua J, Zhang C, Zhao K. Innovative applications of bacteria and their derivatives in targeted tumor therapy. ACS Nano. 2025;19:5077–109. 10.1021/acsnano.4c15237. 39874477 10.1021/acsnano.4c15237

[48] Chang Z, Guo X, Li X, Wang Y, Zang Z, Pei S, et al. Bacterial immunotherapy leveraging IL-10R hysteresis for both phagocytosis evasion and tumor immunity revitalization. Cell. 2025;188(7):1842–57. 10.1016/j.cell.2025.02.002 10.1016/j.cell.2025.02.002 40037354

[49] Peng F, Hu M, Su Z, Hu L, Guo L, Yang K. Intratumoral microbiota as a target for advanced cancer therapeutics. Adv Mater. 2024;36:2405331. 10.1002/adma.202405331. 10.1002/adma.202405331 39054925

[50] Longmore GD. Bacteria in tumors “hit the road” together. Cell. 2022;185:1292–4. 10.1016/j.cell.2022.03.013 35427497 10.1016/j.cell.2022.03.013 PMC9289786

[51] Zhou S, Gravekamp C, Bermudes D, Liu K. Tumour-targeting bacteria engineered to fight cancer. Nat Rev Cancer. 2018;18:727–43. 10.1038/s41568-018-0070-z. 30405213 10.1038/s41568-018-0070-z PMC6902869

[52] Zhang H, Wang Y, Li M, Cao K, Qi Z, Zhu L, et al. A self-guidance biological hybrid drug delivery system driven by anaerobes to inhibit the proliferation and metastasis of colon cancer. Asian J Pharm Sci. 2022;17:892–907. 10.1016/j.ajps.2022.09.003. 36600894 10.1016/j.ajps.2022.09.003 PMC9800957

[53] Kesharwani P, Puri V, Alqahtani T, Al Shmrany H, Gupta G, Goh KW, et al. PEGylated dendrimers for precision cancer therapy: advances in tumor targeting, drug delivery, and clinical translation. Biomater Adv. 2026;179:214493. 10.1016/j.bioadv.2025.214493. 40930026 10.1016/j.bioadv.2025.214493

[54] Attia MF, Anton N, Wallyn J, Omran Z, Vandamme TF. An overview of active and passive targeting strategies to improve the nanocarriers efficiency to tumour sites. J Pharm Pharmacol. 2019;71:1185–98. 10.1111/jphp.13098. 31049986 10.1111/jphp.13098

[55] Xu Y, Wu H, Huang J, Qian W, Martinson DE, Ji B, et al. Probing and enhancing ligand-mediated active targeting of tumors using sub-5 Nm ultrafine iron oxide nanoparticles. Theranostics. 2020;10:2479–94. 10.7150/thno.39560. 32194814 10.7150/thno.39560 PMC7052897

[56] Xu Z, Xie Y, Chen W, Deng W. Nanocarrier-based systems for targeted delivery: current challenges and future directions. MedComm. 2025;6:e70337. 10.1002/mco2.70337. 40859956 10.1002/mco2.70337 PMC12371215

[57] Lin D, Feng X, Mai B, Li X, Wang F, Liu J, et al. Bacterial-based cancer therapy: an emerging toolbox for targeted drug/gene delivery. Biomaterials. 2021;277:121124. 10.1016/j.biomaterials.2021.121124. 34534860 10.1016/j.biomaterials.2021.121124

[58] Liang C, Yang H, Li T, Jiang X, Li X, Gao C, et al. On‐Demand controlled release multi‐drugs delivery system for spatiotemporally synergizing antitumor immunotherapy. Adv Sci. 2025;12:2414233. 10.1002/advs.202414233 10.1002/advs.202414233 PMC11884579 39792614

[59] Lu Y, Mei N, Ying Y, Wang D, Li X, Zhao Y, et al. Bacteria-based nanoprobes for cancer therapy. Int J Nanomed. 2024;19:759–85. 10.2147/IJN.S438164 10.2147/IJN.S438164 PMC10821665 38283198

[60] Li Y, Leng Q, Zhang Y, Lin S, Wen Q, Lu Y, et al. Anaerobic bacteria mediated ‘smart missile’ targeting tumor hypoxic area enhances the therapeutic outcome of lung cancer. Chem Eng J. 2022;438:135566. 10.1016/j.cej.2022.135566.

[61] Li J, Wen Q, Dai J, Wang B, Lu Y, Wu Z, et al. An oral bioactive chitosan-decorated doxorubicin nanoparticles/bacteria bioconjugates enhance chemotherapy efficacy in an in-situ breast cancer model. Int J Biol Macromol. 2024;267:131428. 10.1016/j.ijbiomac.2024.131428. 38583834 10.1016/j.ijbiomac.2024.131428

[62] Chen XX, Hou MJ, Wang WX, Tan M, Tan ZK, Mao GJ, et al. ATP-responsive near-infrared fluorescent nanoparticles for synergistic chemotherapy and starvation therapy. Nanoscale. 2022;14:3808–17. 10.1039/D1NR07233A 35191447 10.1039/d1nr07233a

[63] Xiao Y, Pan T, Da W, Liu Y, Chen S, Chen D, et al. Aptamer-drug conjugates-loaded bacteria for pancreatic cancer synergistic therapy. Sig Transduct Target Ther. 2024;9:272. 10.1038/s41392-024-01973-3. 10.1038/s41392-024-01973-3 PMC11471780 39397032

[64] Ghosh S, Sarkar B, Kaushik A, Mostafavi E. Nanobiotechnological prospects of probiotic microflora: synthesis, mechanism, and applications. Sci Total Environ. 2022;838:156212. 10.1016/j.scitotenv.2022.156212. 35623529 10.1016/j.scitotenv.2022.156212

[65] Jin A, Wang Y, Lin K, Jiang L. Nanoparticles modified by polydopamine: working as drug carriers. Bioact Mater. 2020;5:522–41. 10.1016/j.bioactmat.2020.04.003. 32322763 10.1016/j.bioactmat.2020.04.003 PMC7170807

[66] Zeng C, Hua S, Zhou J, Zeng T, Chen J, Su L, et al. Oral microalgae‐based biosystem to enhance irreversible electroporation immunotherapy in hepatocellular carcinoma. Adv Sci. 2025;12:2409381. 10.1002/advs.202409381 10.1002/advs.202409381 PMC12005737 39874200

