# Supplementary material for: Bacteria-powered LA@CaDGP biomotor: a multi-modal weapon integrating calcium overload, chemotherapy, and starvation for breast cancer therapy
Source: J Nanobiotechnology. 2026 Jan 6;24:112. doi: 10.1186/s12951-025-03968-w (PMC12870040; doi:10.1186/s12951-025-03968-w)
Supplement: Supplementary file 1 — Supplementary Material 1 [file 12951_2025_3968_MOESM1_ESM.docx]

**Supplementary Figures**

**Bacteria-Powered LA@CaDGP Biomotor: A Multi-Modal Weapon Integrating Calcium Overload, Chemotherapy, and Starvation for Breast Cancer Therapy**

Jingrong Huang^a,#^, Yongcheng Tang^b,#^, Kewei Xiang^a,#^, Biqiong Wang^a^, Jia Wang^a^, Yun Lu^a^, Yue Li^c^, Hongjun Deng^a^, Tao Li^a^, Kang Xiong^a^, Qinglian Wen^d*^, Shaozhi Fu^a,e*^

*^a^Department of Oncology, The Affiliated Hospital, Southwest Medical University, Luzhou, Sichuan, 646000, PR China*

*^b^Department of General Surgery (Hepatopancreatobiliary Surgery), The Affiliated Hospital, Southwest Medical University, Luzhou, Sichuan, 646000, PR China*

*^c^Medicine and health Klinikum rechts der Isar, Technical University of Munich, Ismaningerstr. 22, 81675 München, Germany*

*^d^Department of Radiation Oncology, Cancer Center, West China Hospital, Sichuan University, Chengdu, Sichuan,610041, PR China*

*^e^Nuclear Medicine and Molecular Imaging Key Laboratory of Sichuan Province, Luzhou, Sichuan, 646000, PR China*


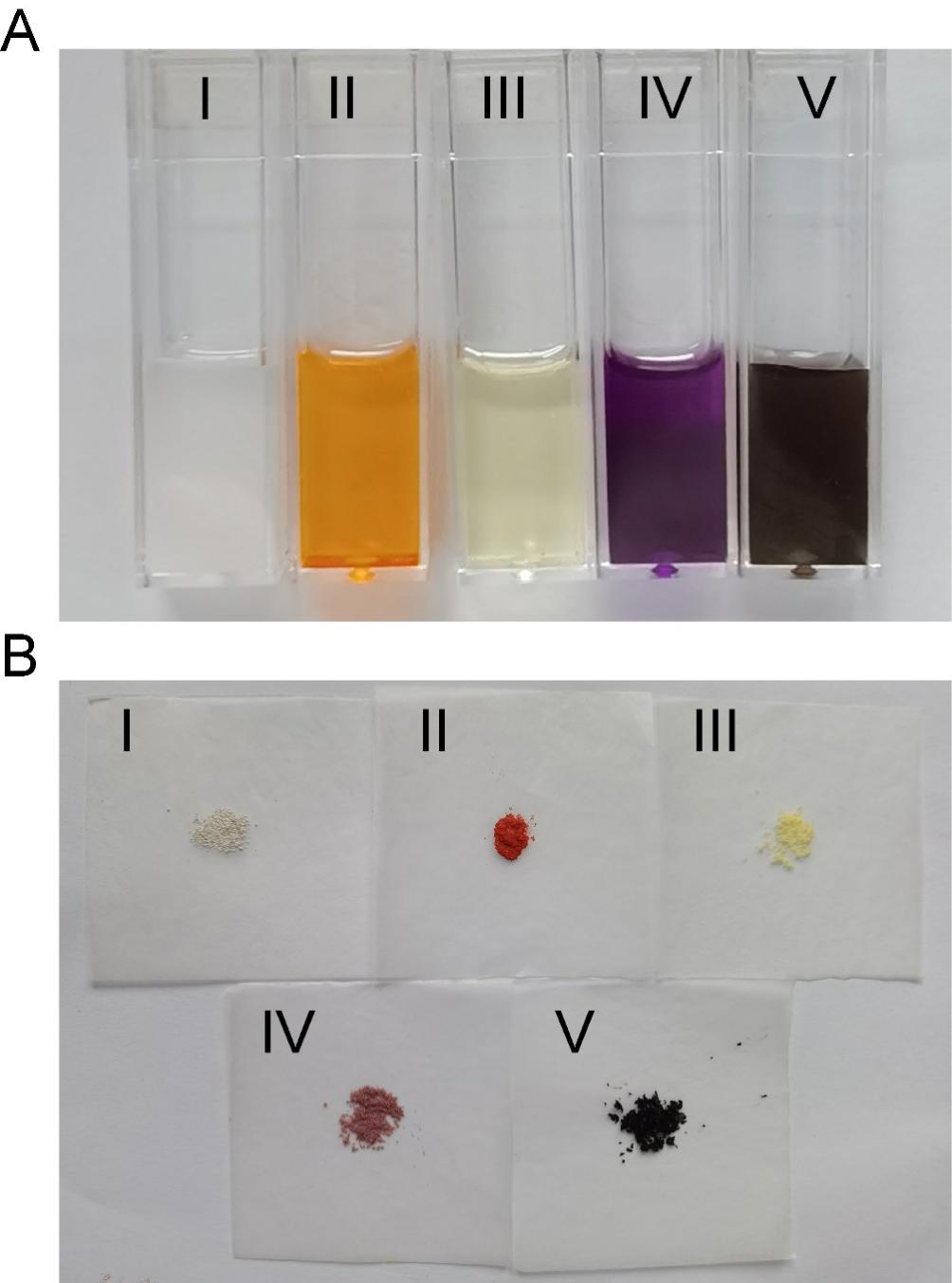


**Figure S1.** Photo of various drug solutions (A) and powders (B). (I: CaCO_3_, II: DOX, III: GOD, IV: CaDG, V: CaDGP)


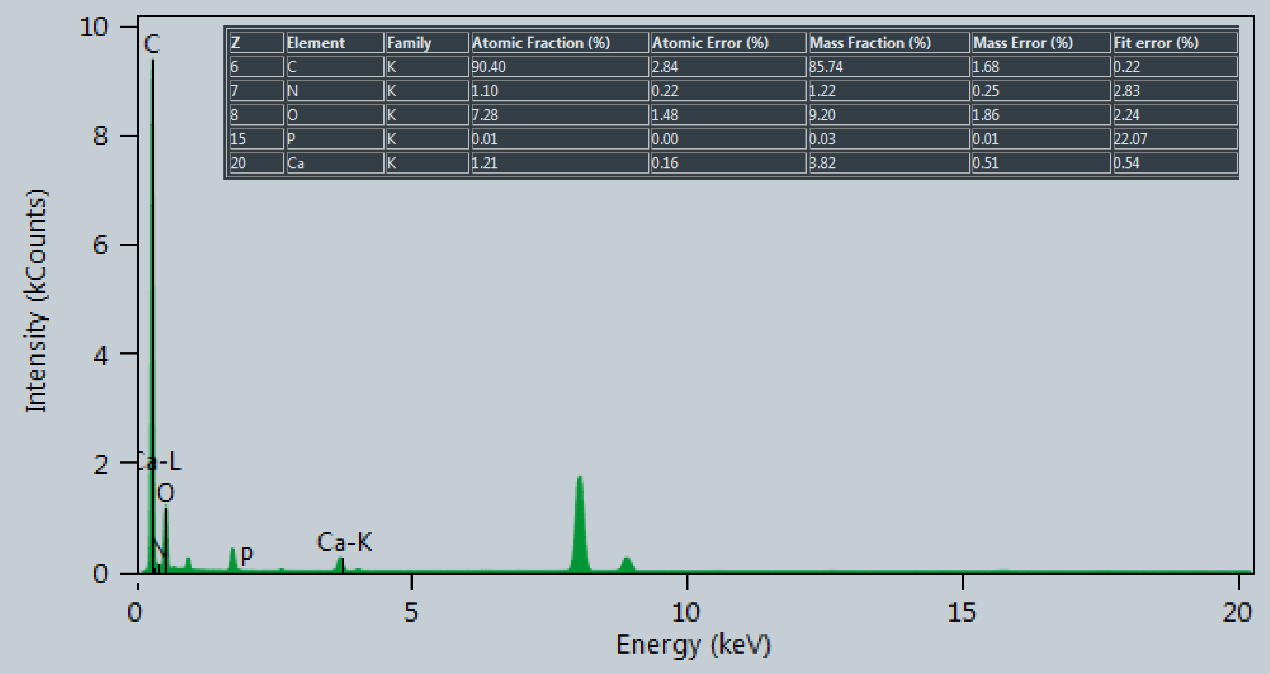


**Figure S2.** The result of EDS analysis of the CaDGP nanoparticles.


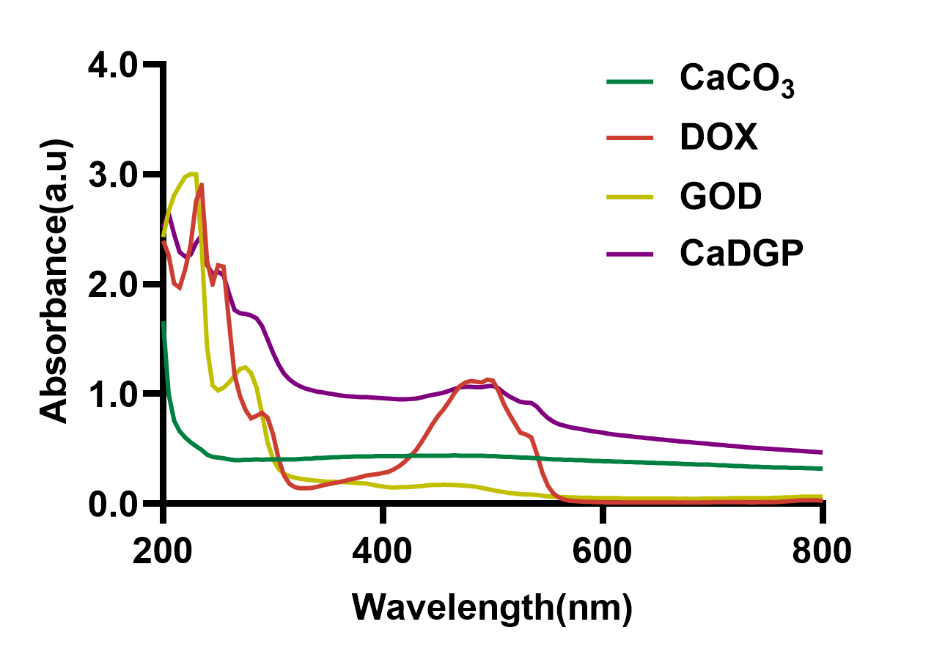


**Figure S3.** UV-vis spectra of the CaCO_3_, DOX, GOD, and CaDGP solutions.

**
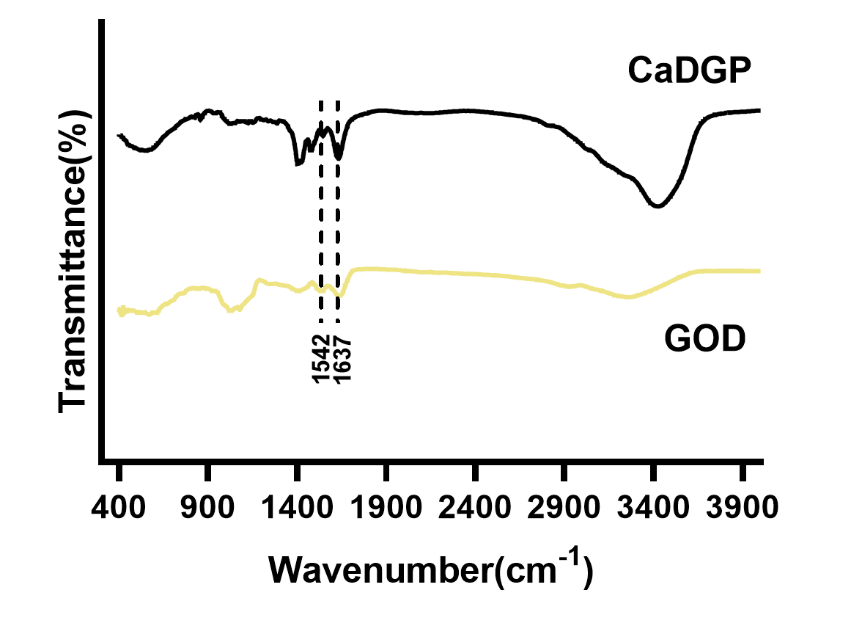
**

**Figure S4.** FT-IR spectra of GOD and CaDGP NPs.


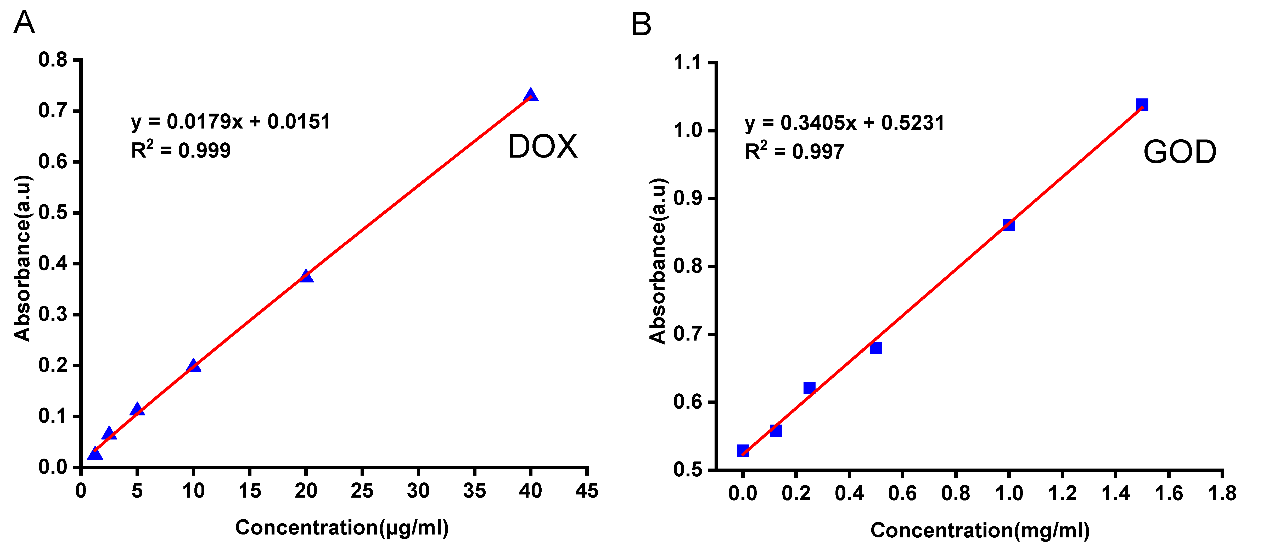


**Figure S5.** The standard curves of (A) DOX at 480 nm and (b) GOD at 275 nm.


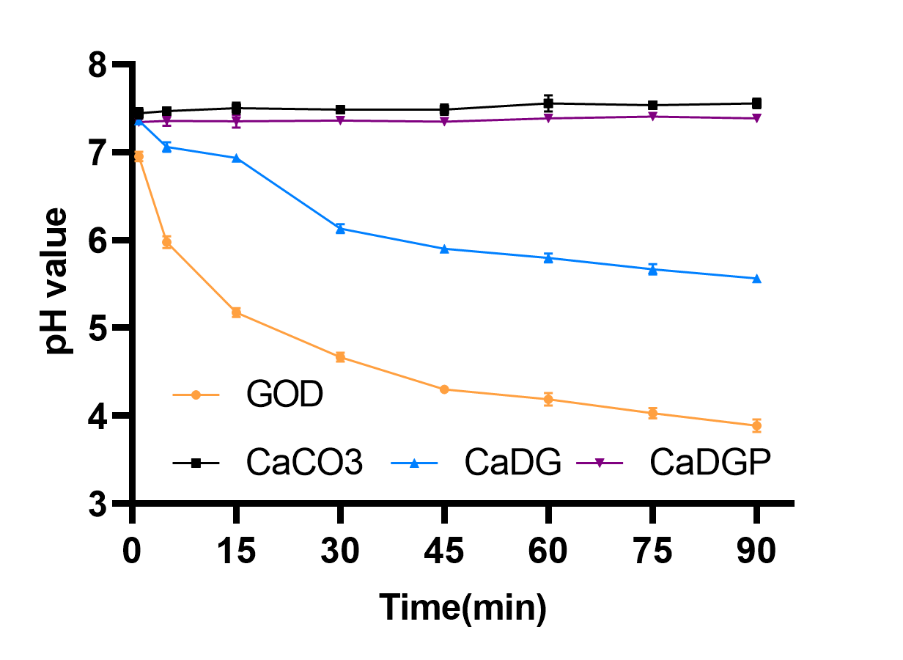


**Figure S6.** The changes of pH values in glucose solutions (1.0 mg/mL) after adding different drugs.


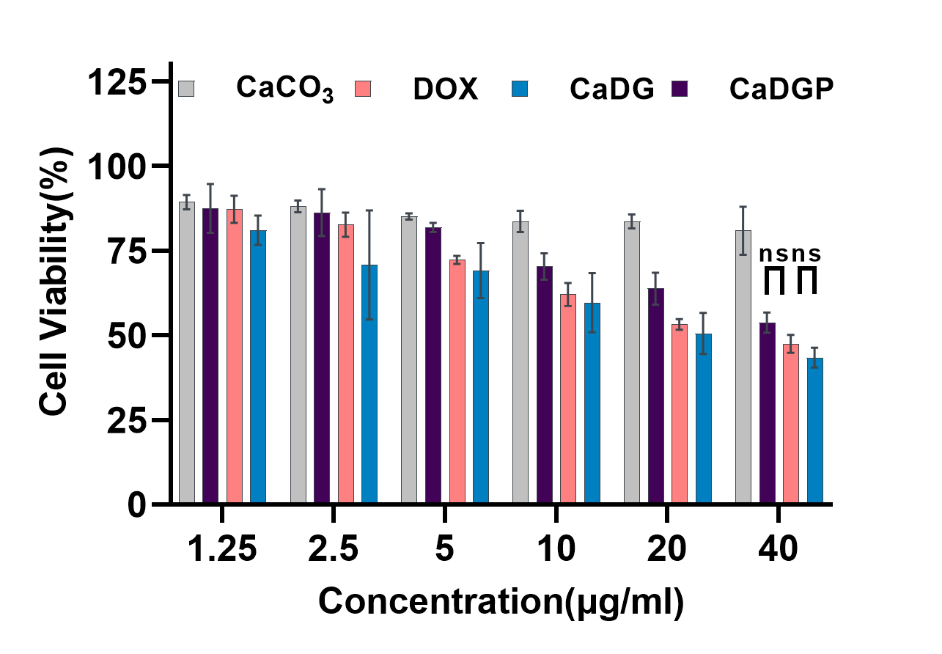


**Figure S7.** In vitro cytotoxicity of CaCO_3_, DOX, CaDG, and CaDGP on 4T1 cells at pH 7.4, ns: no statistical significance.


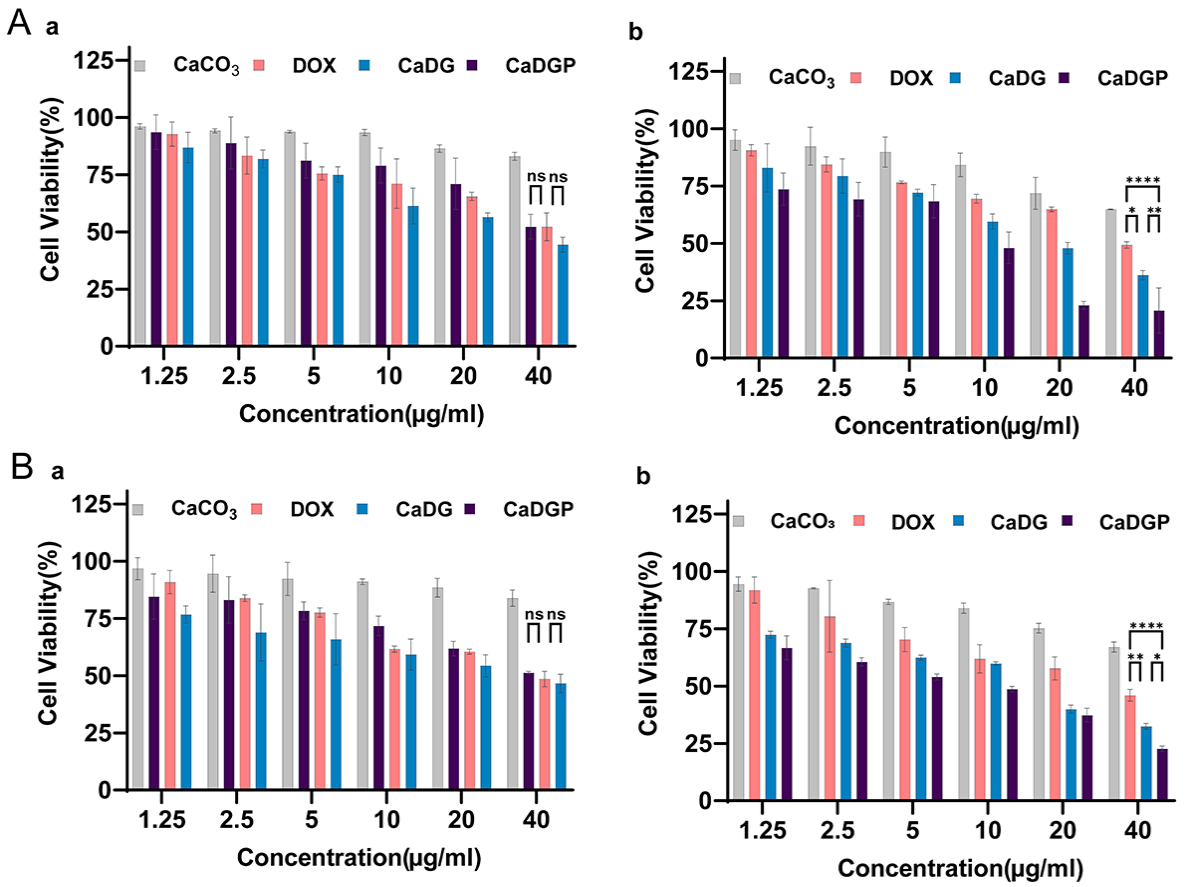


**Figure S8.** In vitro cytotoxicity of CaCO_3_, DOX, CaDG, and CaDGP. (A) Cell viability of A549 cells at pH 7.4 (a) and pH 6.5 (b), (B) cell viability of CT26 cells at pH 7.4 (a) and pH 6.5 (b). ns: no statistical significance, **P*<0.05, ***P*<0.01, *****P*<0.0001.


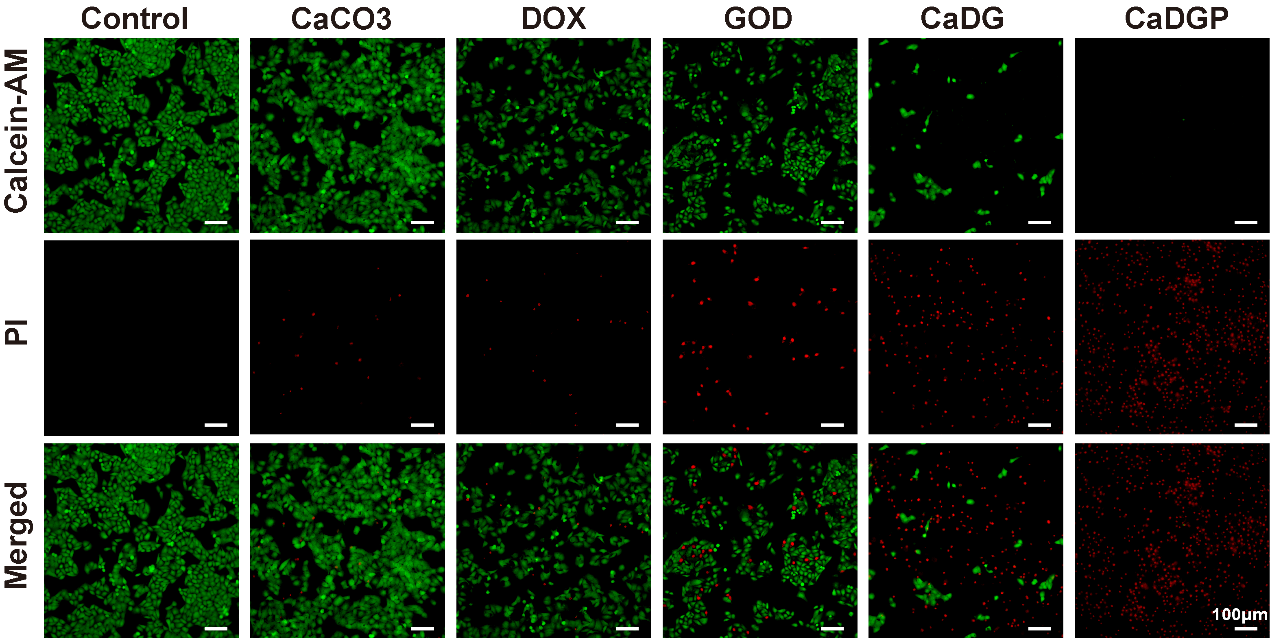


**Figure S9.** Live/dead staining with Calcein-AM (green, live cells) and PI (red, dead cells) of A549 cells in various treatments, scale bar: 100 μm.


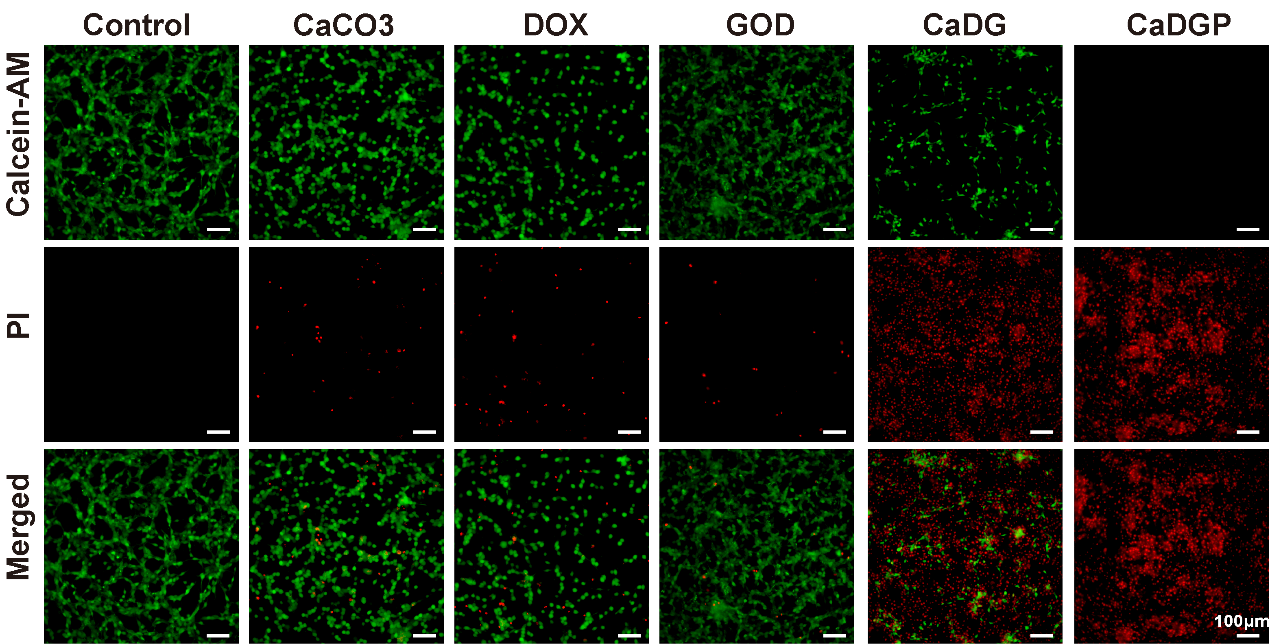


**Figure S10.** Live/dead staining with Calcein-AM (green, live cells) and PI (red, dead cells) of CT26 cells in various treatments, scale bar: 100 μm.


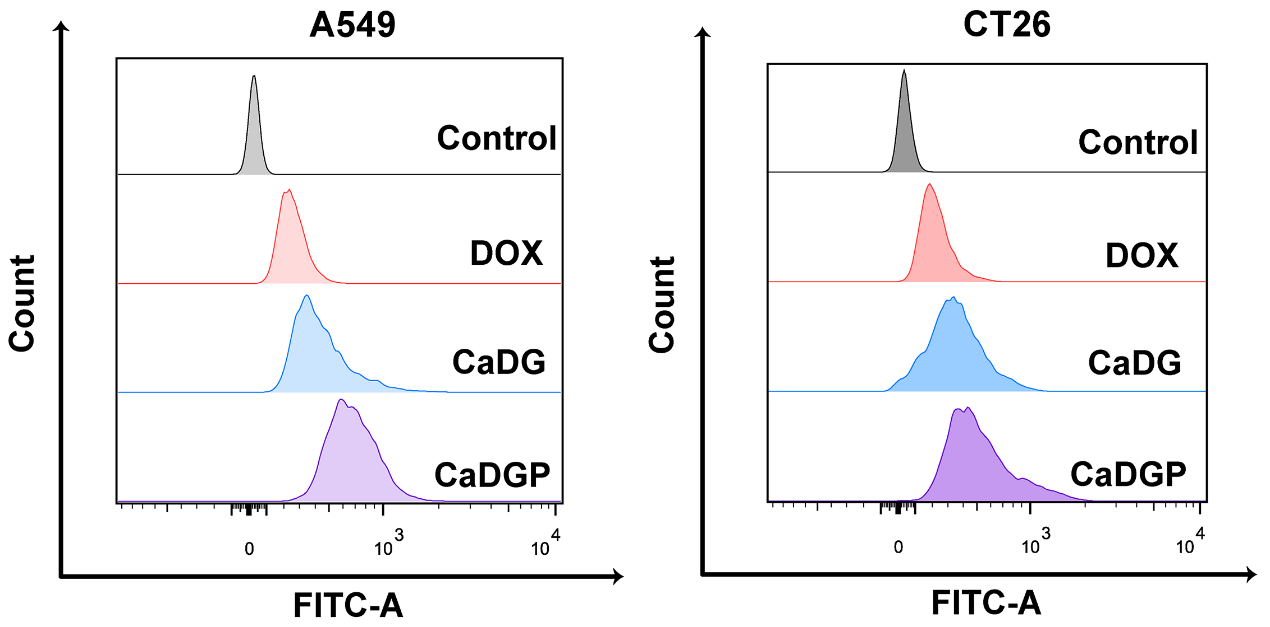


**Figure S11.** Quantitative analysis of fluorescence intensity of DOX within A549 cells and CT26 cells.


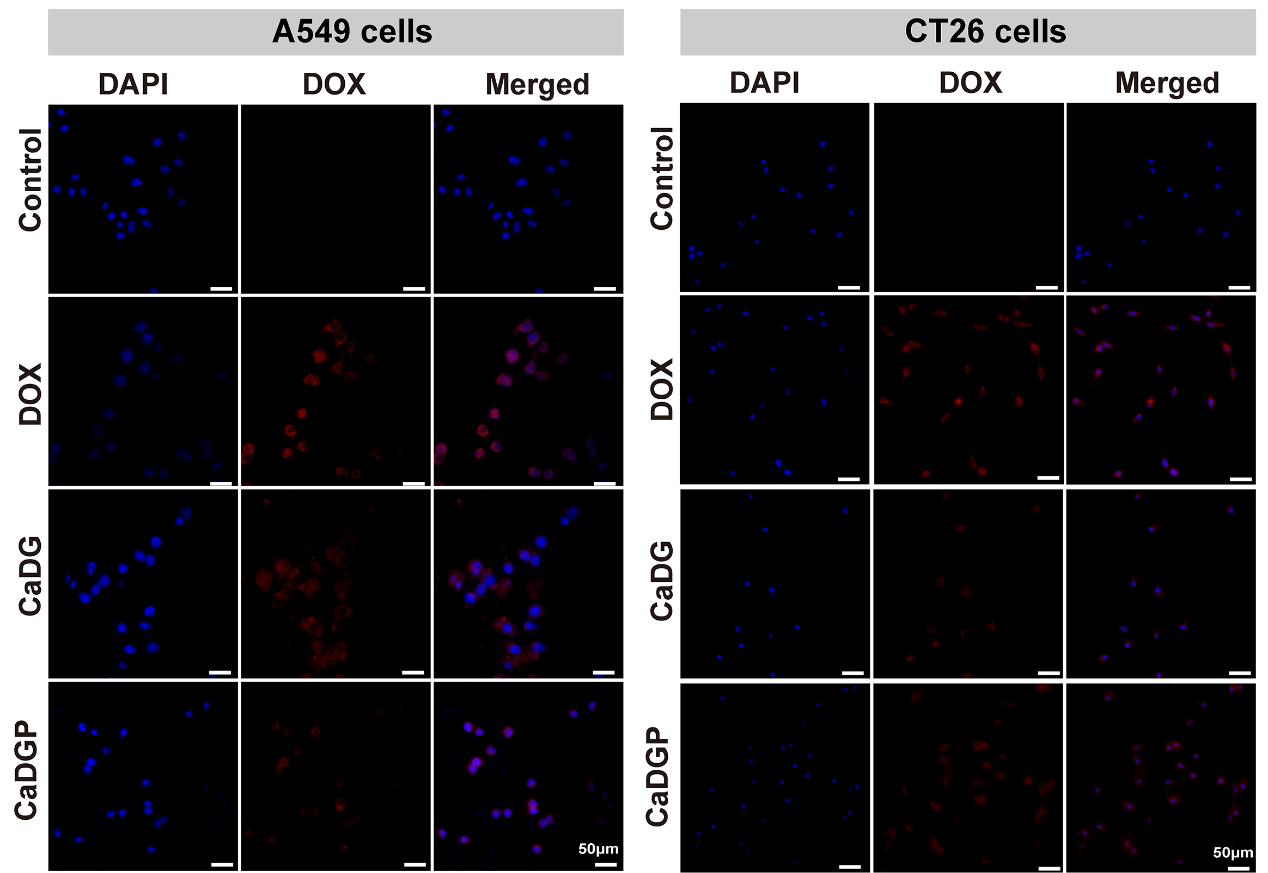


**Figure S12.** Fluorescence images depict the cell uptake of DOX, CaDG, and CaDGP by A549 and CT26 cells, scale bar: 50 μm.


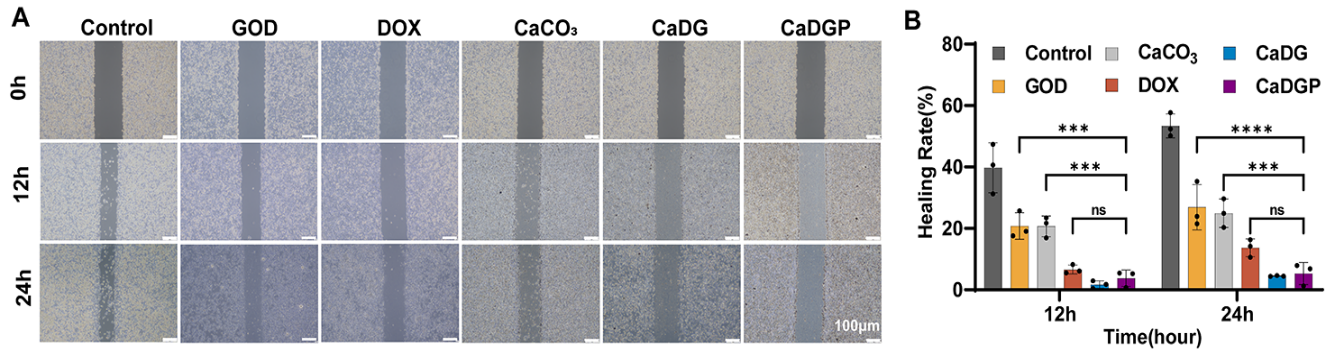


**Figure S13.** Migration inhibition assay. (A) Photographs of wound healing on A549 cells taken at different time points, scale bar: 100 μm. (B) The healing rate of each group. ns: no statistical significance, ****P*<0.001, *****P*<0.0001.


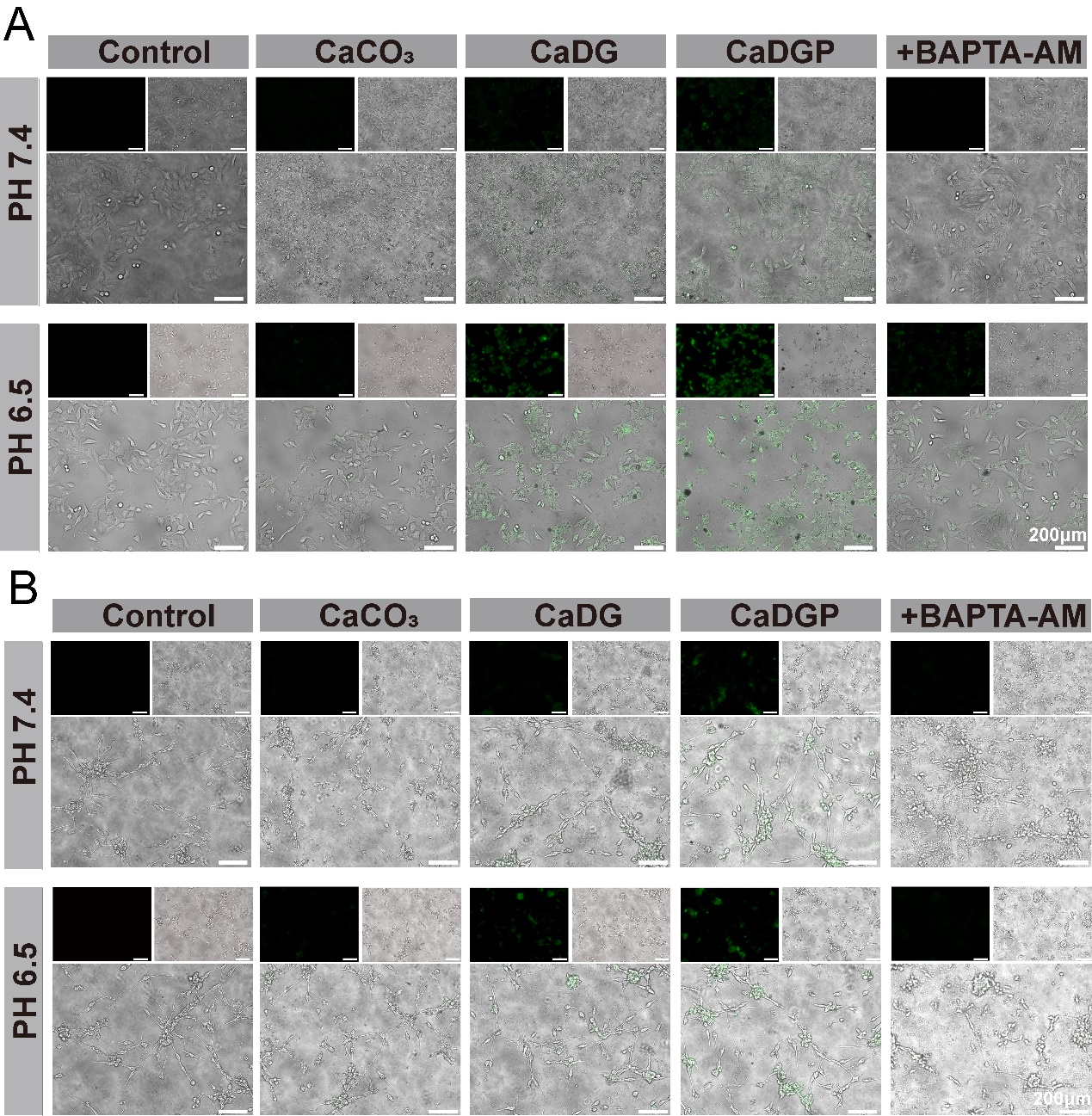


**Figure S14.** Fluorescence images of intracellular uptake of Ca^2+^ in (A) A549 cells and (B) CT26 after different treatments at pH 7.4 and 6.5, scale bar: 200 μm.


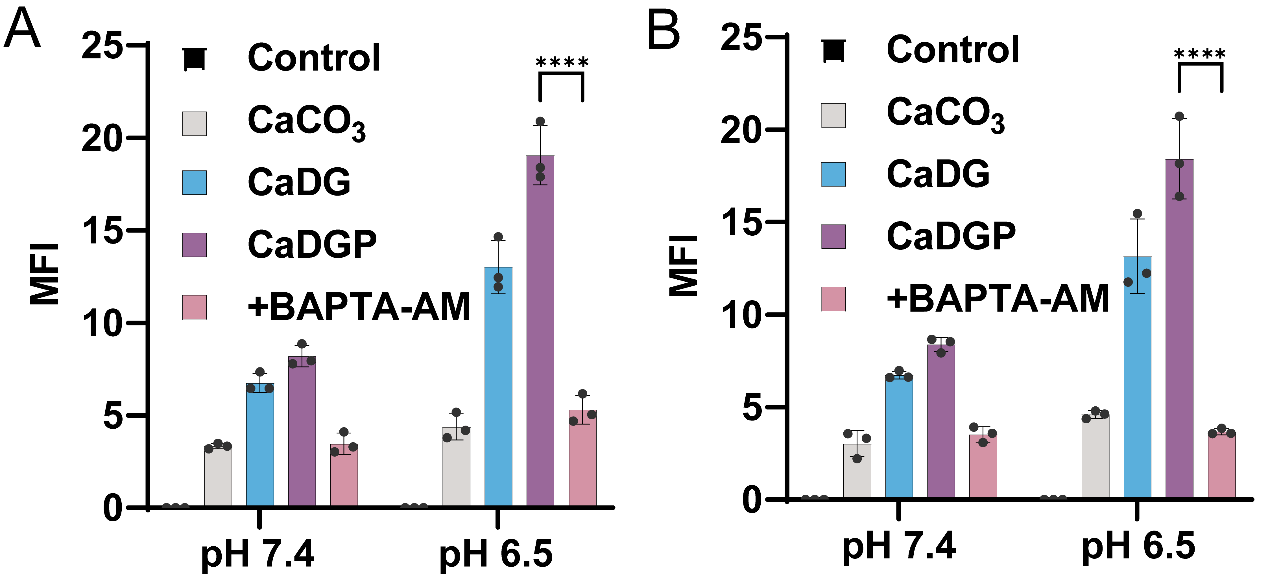


**Figure S15.** Quantitative fluorescence intensity of intracellular uptake of Ca^2+^ in (A) A549 cells and (B) CT26 cells. *****P*<0.0001.


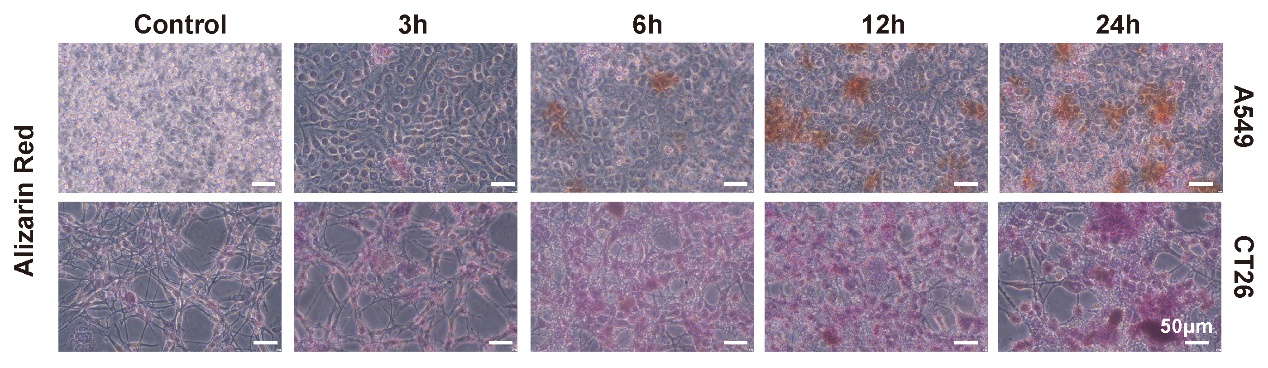


**Figure S16.** Alizarin Red staining of A549 cells and CT26 cells, scale bar:50 μm.


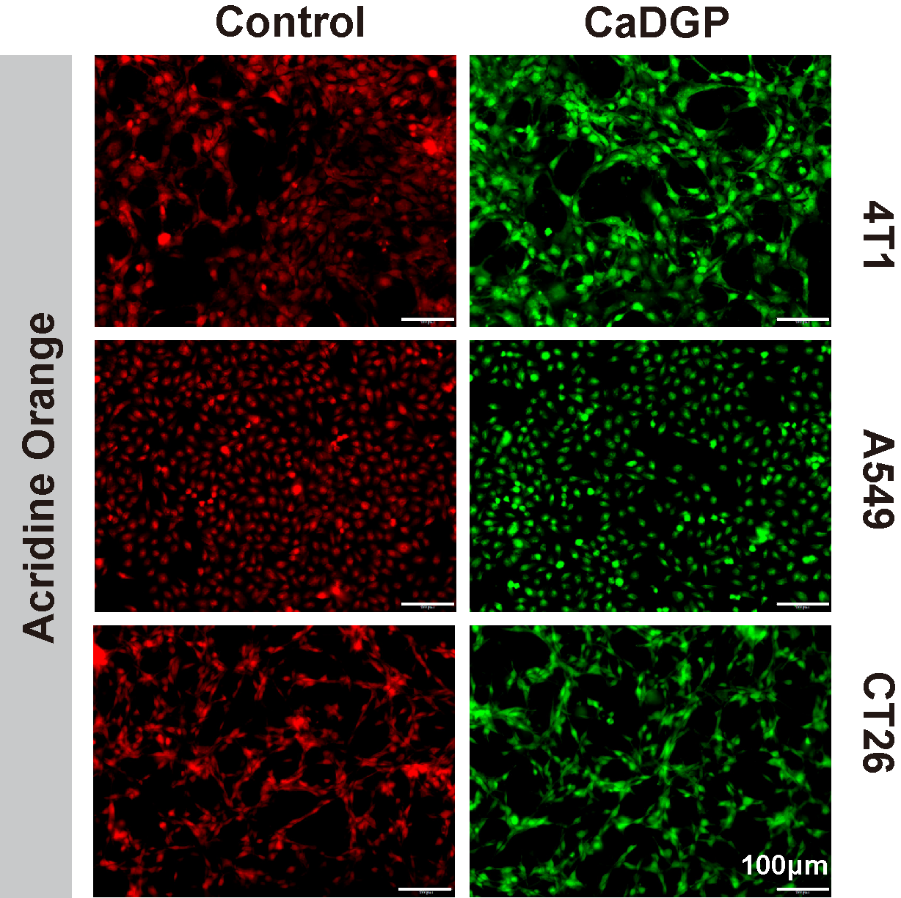


**Figure S17.** Fluorescence images of AO-stained on 4T1 cells, A549 cells, and CT26 cells, scale bar: 100 μm.


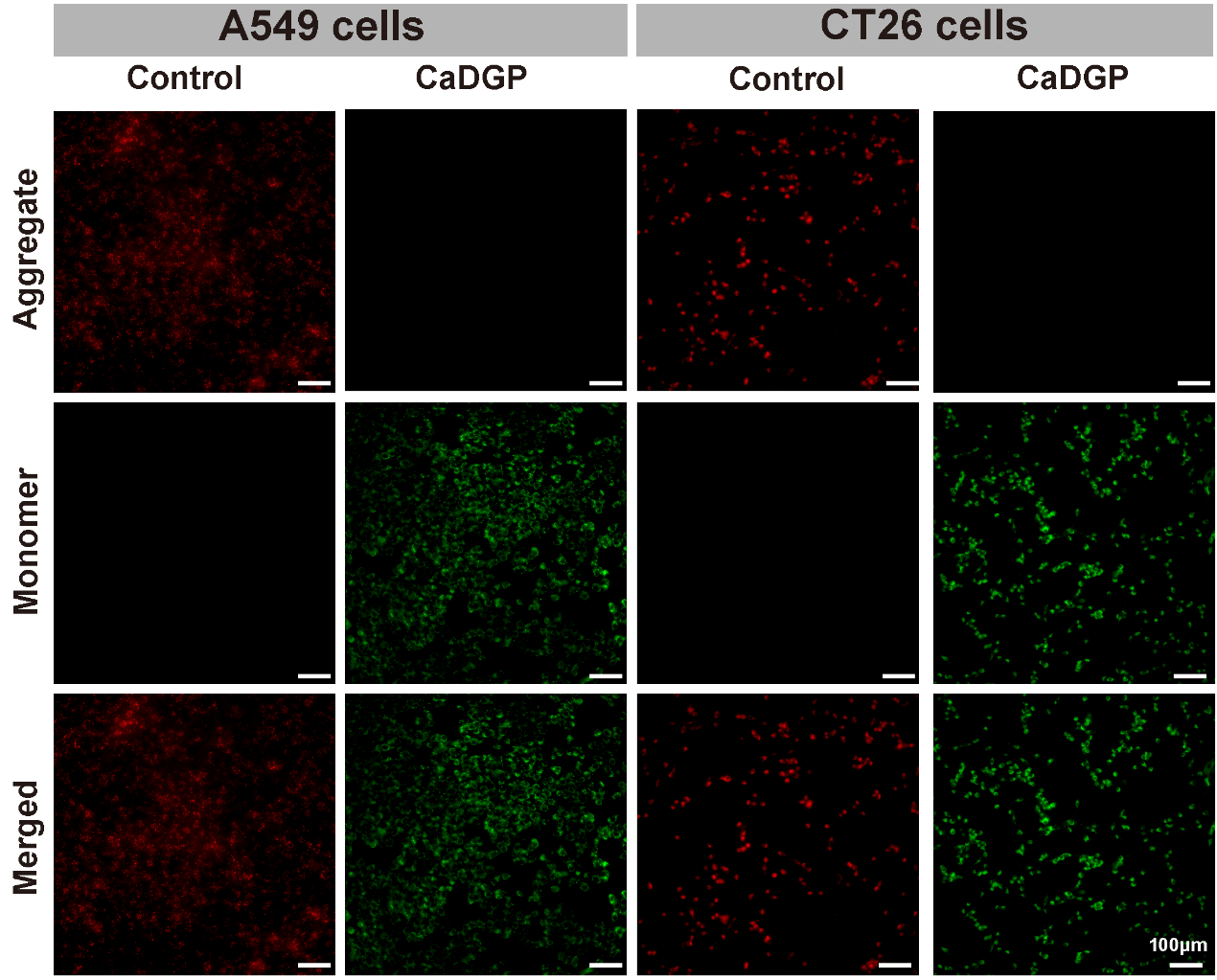


**Figure S18.** Fluorescence images of JC-1-stained A549 cells and CT26 cells, scale bar: 100 μm.


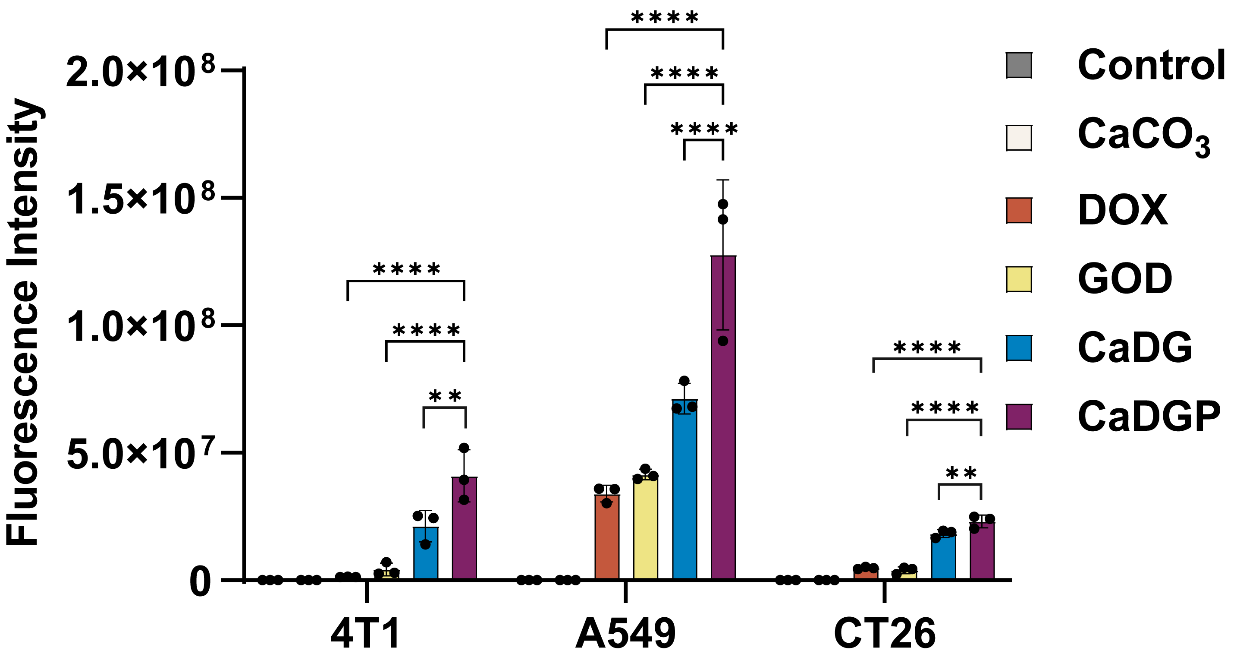


**Figure S19.** The quantitative fluorescence intensity of DCFH-DA in 4T1, A549, and CT26 cells. ns: no statistical significance, **P*<0.05, ***P*<0.01, *****P*<0.0001.


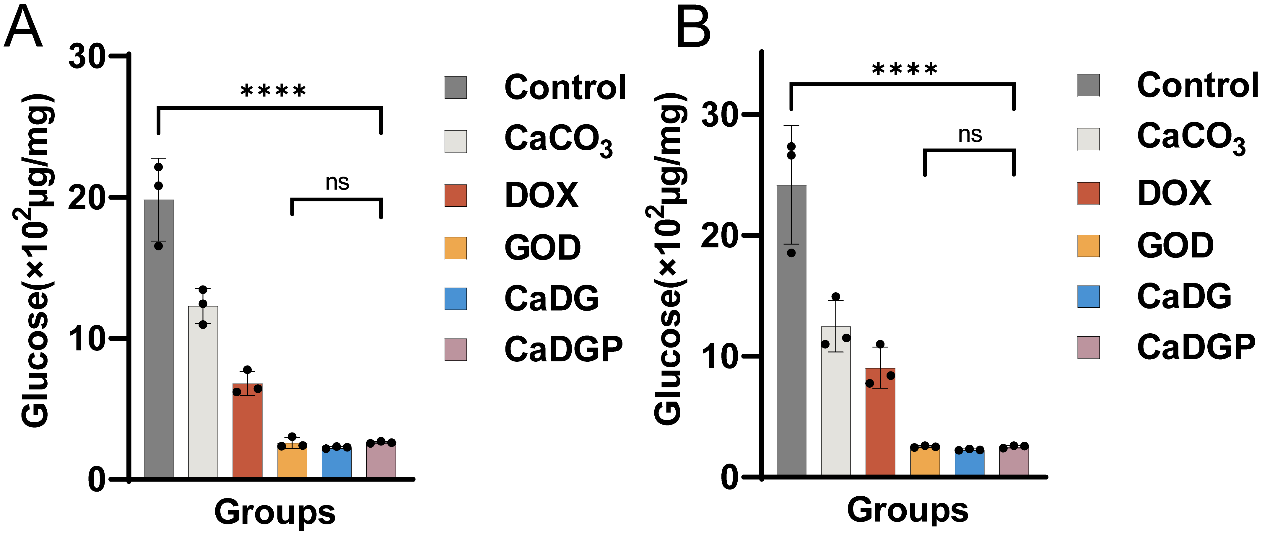


**Figure S20.** Glucose content in (A) A549 cells and (B) CT26 cells after treated with various drugs. ns: no statistical significance, *****P*<0.0001.


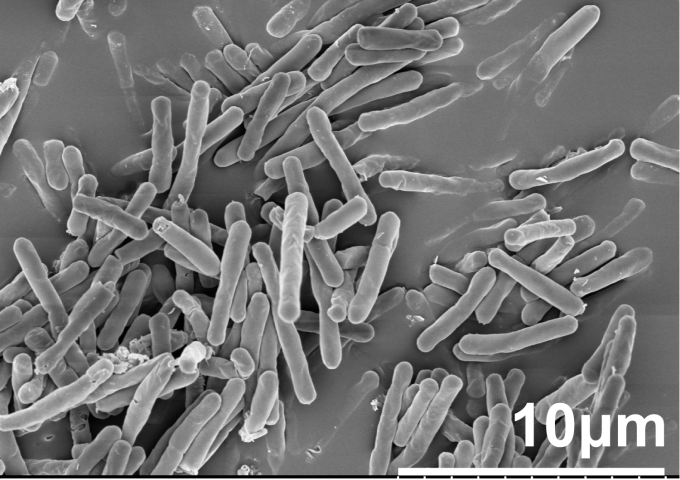


Figure S21. SEM image of the naked LA. (Scar bar: 10 µm)


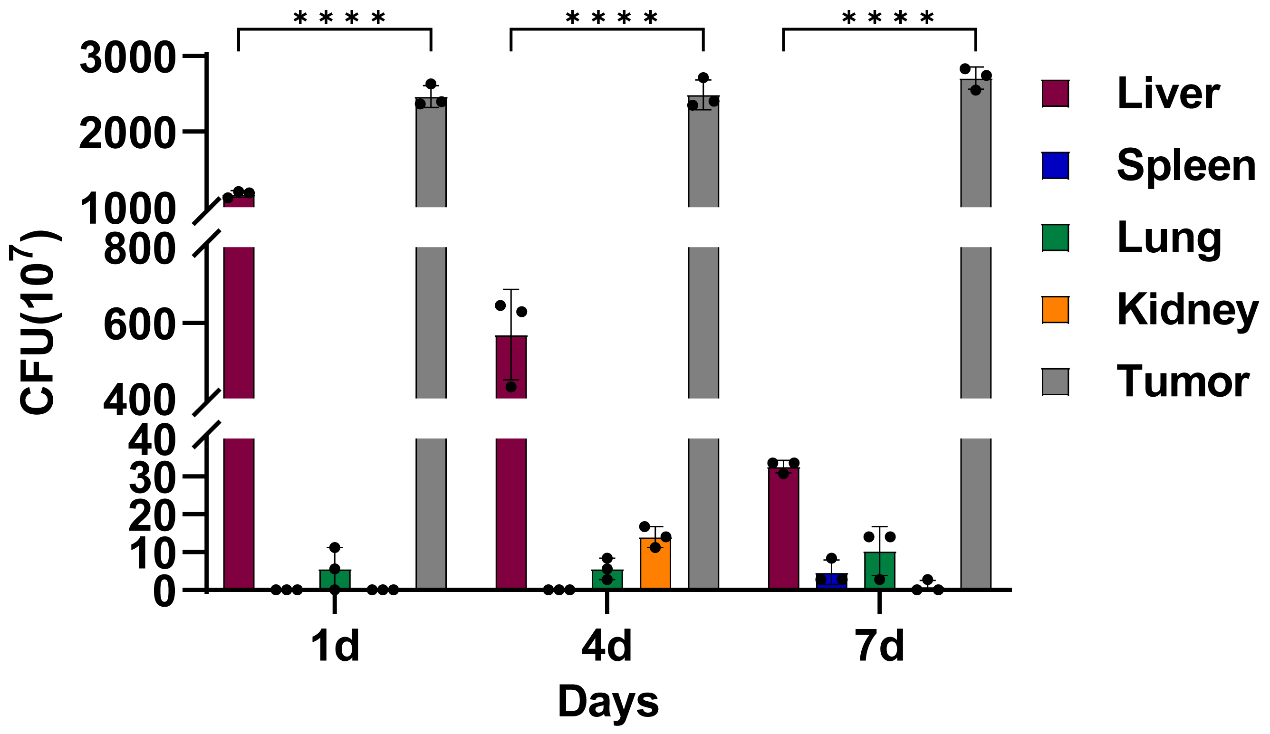


**Figure S22.** The number of LA in the liver, spleen, lung, kidney, and tumor after treated with LA@CaDGP orally. *****P*<0.0001.

**
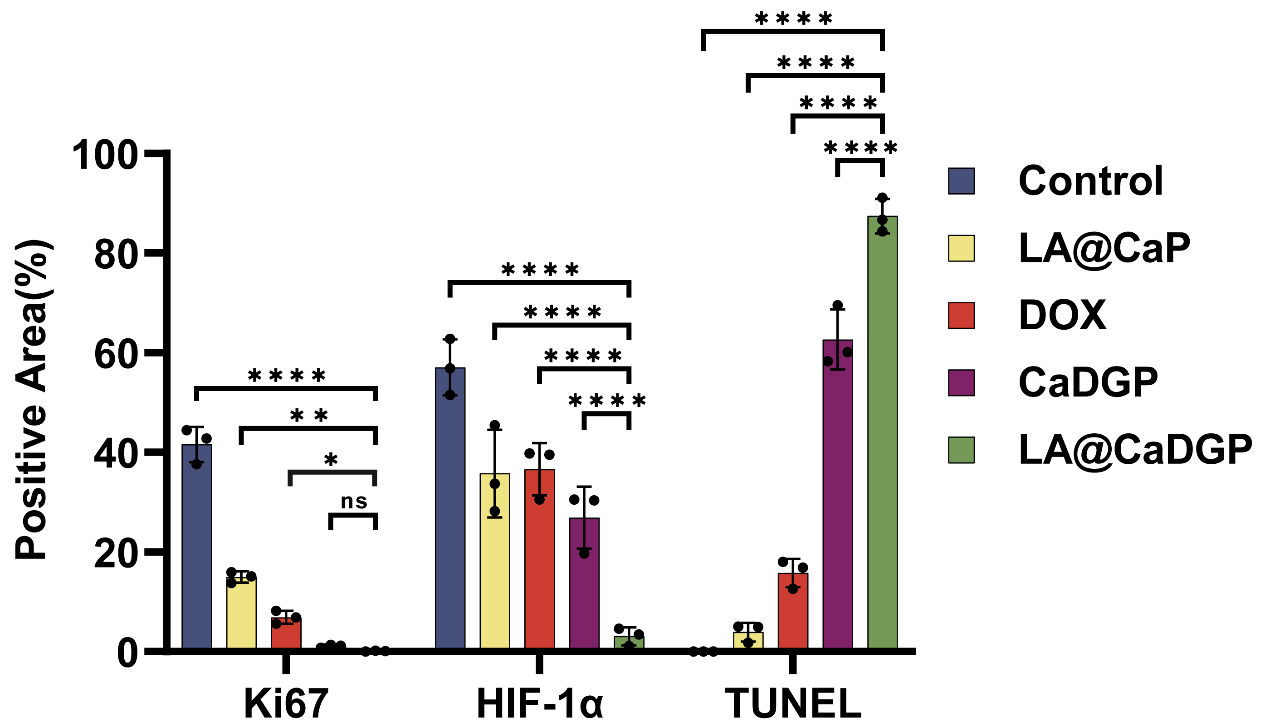
**

**Figure S23.** Quantitative analysis of the Ki-67, HIF-1α, and TUNEL in tumors. ns: no statistical significance, **P*<0.05, ***P*<0.01, *****P*<0.0001.


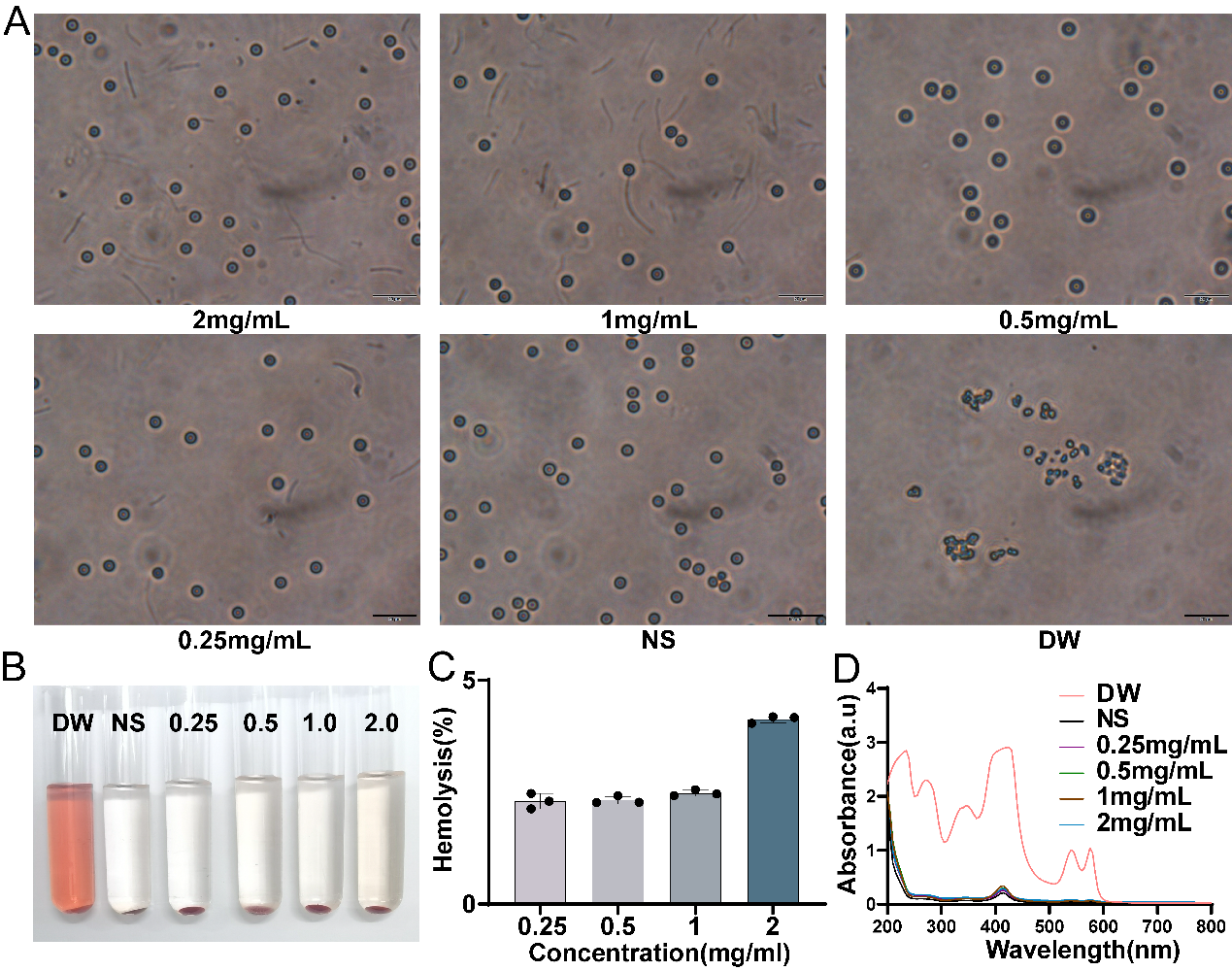


**Figure S24.** In vitro hemolysis assay. (A) Microscopic photographs showing representative erythrocyte cultures with various concentrations of LA@CaDGP; NS (normal saline): negative control; DW (distilled water): positive control. (B) Photographs demonstrating hemolysis. (C) Hemolysis rate in each group. (D) UV-Vis absorption spectra in each group.
